# Multigram Synthesis of Pure HMF and BHMF

**DOI:** 10.1021/acs.oprd.2c00196

**Published:** 2022-09-09

**Authors:** Giacomo Trapasso, Giovanna Mazzi, Beatriz Chícharo, Mattia Annatelli, Davide Dalla Torre, Fabio Aricò

**Affiliations:** Department of Environmental Sciences Informatics and Statistics, Ca’ Foscari University of Venice, Via Torino, 155, 30170 Mestre, Venezia, Italy

**Keywords:** green chemistry, biorefinery, scale-up reaction, HMF, dimethyl carbonate, green metrics

## Abstract

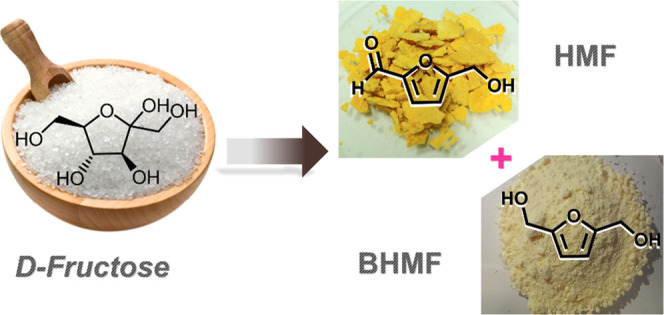

5-Hydroxymethylfurfural (HMF) is a bio-based platform chemical that can be used as a building block to produce several compounds with diverse applications. Even though HMF synthesis holds promise for a greener future, the current state of technology and the high production cost limit its competitiveness on an industrial scale. In this prospect, we have developed a multigram-scale procedure for HMF by reacting d-fructose with Purolite CT275DR—an acidic resin—in a dimethyl carbonate (DMC)/tetraethyl ammonium bromide (TEAB) biphasic system. Reactions performed in an autoclave for 2 h at 110 °C using up to 40 gram of d-fructose resulted in an overall HMF yield of 70%. HMF was purified by a custom-made procedure leading to ca 50% of the pure crystalline product; meanwhile, the residual HMF-rich oil was directly reduced to bis(hydroxymethyl)furan (BHMF). Green metrics and the Ecoscale algorithm were used to evaluate the sustainability of the herein-proposed procedure in comparison with previously reported works.

## Introduction

The inevitable reduction of fossil-derived resources due to the continuous exploitation of petroleum and coal feedstocks, together with the incumbent climate crisis, has encouraged the scientific community to focus on more bio-based alternatives. As a result, biorefinery was developed with the aim to discover and produce alternative substrates that can economically substitute petroleum-based compounds, consequently reducing greenhouse gas emissions.^1^

Among the various molecules derived from renewables investigated as building blocks to produce complex and useful value-added compounds,^2^ 5-hydroxymethylfurfural (HMF) is one of the most studied due to its versatile reactivity.^3^ HMF can be subjected to oxidation,^4,5^ hydrogenation,^5,6^ hydrolysis,^7^ esterification,^8^ etc., leading to a variety of chemicals such as furan-2,5-dicarbaldehyde (FFC),^2,4^ 2,5-furandicarboxylic acid (FDCA)^9,10^ and its esters,^11^ bis(hydroxymethyl)furan (BHMF) and its derivatives,^12^ 5,5′-oxy(bismethylene)-2-furaldehyde (OBMF),^13^ 2,5-dimethyl furan (DMF),^6^ and levulinic acid.^9^ Each of these compounds has demonstrated a wide array of uses as reaction intermediates, monomers for biopolymers, or additives for fuels.^2^ HMF, in particular, has exhibited potential applications in the pharmaceutical, food, materials, and chemical industries.^14,15^ Nevertheless, due to its intrinsic thermodynamic instability, HMF production often leads to side reactions, e.g., rehydration to formic acid and levulinic acid,^16,17^ oligomerization,^18^ cross-polymerization, and carbohydrate formation,^5^ which promote the synthesis of up to 36 side products^5^ together with the formation of unwanted humins.

**Figure 1:**
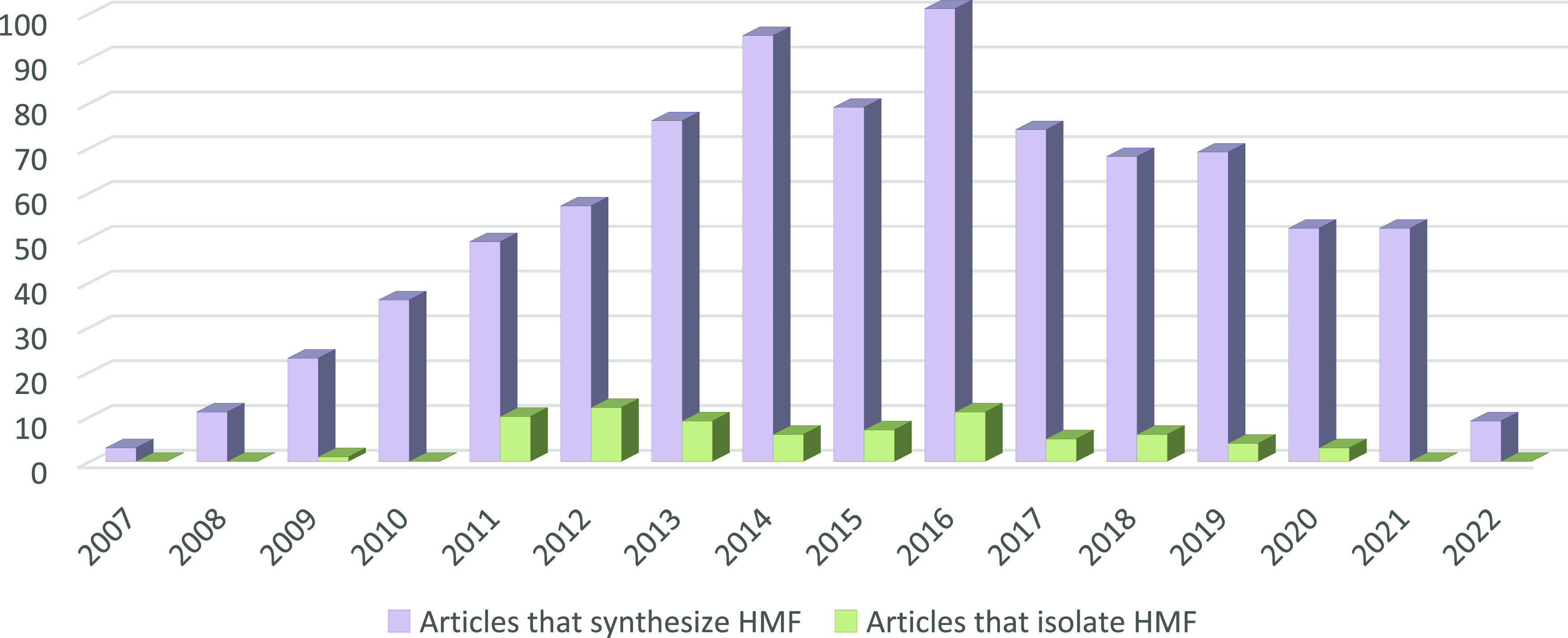
Number of articles on HMF synthesis and isolation published per year.

Throughout the years, a variety of synthetic routes to HMF have been reported in the literature, with the acid-catalyzed triple dehydration of d-fructose being the most commonly employed.^2,14^ Starting from 2007—when the concept of biorefining emerged—research articles focusing on HMF synthesis followed an evident linear trend, peaking in 2016 (Figure 1). Different procedures were reported encompassing conventional heating in batch, autoclave under pressure, but also microwave- and sonification-mediated reactions.^15^ The use of high temperatures^19^ and non-aqueous media^15^ was explored so as to reduce side reactions and increase both yield and selectivity.

Even though rapid advances are being made in enhancing the efficiency of HMF synthesis, its economical and sustainable viable industrialization is far from happening due to unresolved issues related to product isolation and its stability.^16,20,21^ These challenges are quite obvious considering that among all of the reported procedures less than 10% address HMF isolation and/or purification from the reaction mixture (Figure 1). As a matter of fact, most literature works estimate HMF yield, conversion, and selectivity using different techniques, i.e., HPLC, NMR, and GC.^9^ In addition, 5-hydroxymethylfurfural synthesis is generally conducted in small quantities, which does not facilitate its industrial production and economic development.^2^ Therefore, it is of paramount importance to develop an effective large-scale procedure for this bio-based platform chemical with excellent selectivity and high yields in a sustainable way to ultimately decrease its production cost but also to boost the upgrading of HMF to other industrially appealing derivatives.^2^

**Scheme 1:**
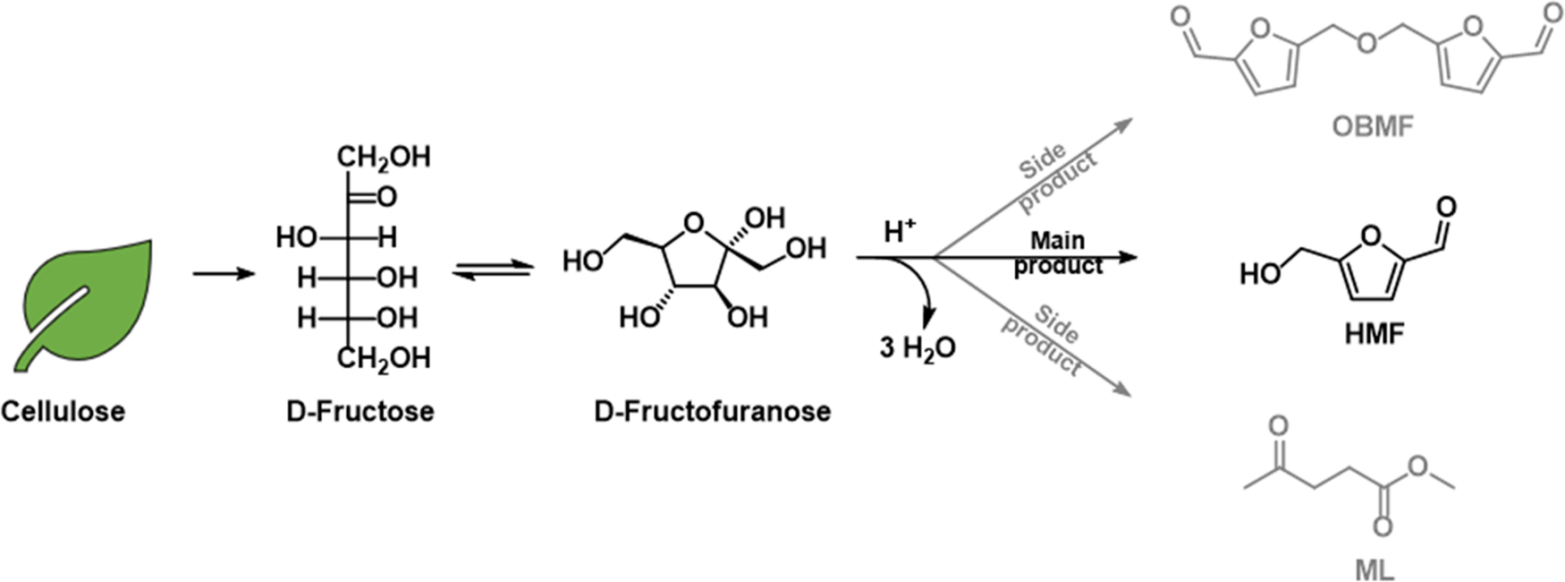
Production of HMF Crystals from D-Fructose; OBMF and Methyl Levulinate (ML) Can Be Obtained as Side Products

In this view, the present work focuses on developing a scalable high-yielding procedure for HMF from d-fructose (up to 40 grams) using a dimethyl carbonate (DMC)/tetraethyl ammonium bromide (TEAB) biphasic system and recycling the excess reagents (Scheme 1).

The synergic action of TEAB and DMC, acting as co-solvents, allowed the complete dissolution of D-fructose and the easy extraction of HMF from the reaction mixture, respectively.^14^ The desired product was recovered in *ca* 50% yield as a pure crystalline powder from the crude mixture adopting a custom-made crystallization procedure^22^ and using non-toxic solvents. The residual HMF-rich oil was directly reduced to bis(hydroxymethyl)furan (BHMF), allowing for the final isolation of two furanic bio-based platform chemicals, i.e., HMF and BHMF, with a simple procedure.

Furthermore, to investigate the environmental impact and the greenness of this approach, a variety of green metrics, such as *E*-factor (*E*-f), atom economy (AE), reaction mass efficiency (RME), mass recovery parameter (MRP), and process mass intensity (PMI), were calculated and compared to the ones of previously published works on HMF synthesis, which addressed product purification and/or scale-up of the reaction.

## Results and Discussion

### HMF Synthesis in Autoclave

In a typical reaction, d-fructose was dissolved in a DMC/TEAB biphasic system in the presence of a heterogeneous acid catalyst (10% by weight). The synthesis was carried out at 110 °C for 2 h in a stainless-steel autoclave under the generated autogenous pressure (Tables 1 and 2).

**Table 1:** Comparison of Different Heterogeneous Acidic Catalysts in the Synthesis of HMF^a^

		selectivity %^b^		b
#	acidic catalyst	HMF	OBMF	yield %
1	Amberlyst-15	98	2	64
2	Amberlyst-36	98	2	65
3	Purolite CT151	94	6	66
4	Purolite CT269	97	3	67
5	Purolite CT275	96	4	63
6	Purolite CT275DR	98	2	73

a Reaction conditions: 3.75 g of d-fructose was dissolved in 30 mL of DMC/TEAB (20% wt.) in the presence of a heterogeneous acidic catalyst (10% wt.), in an autoclave at 110 °C for 2 h.

b Estimated via ^1^H-NMR.

**Table 2:** Synthesis of HMF Employing DMC/TEAB as a Solvent System in an Autoclave^a^

					selectivity^b^ %		
#	DMC (mL)	TEAB (% wt.)	[H^+^] (% wt.)	*t* (h)	HMF	OBMF	yield^b^ (%)
1	80	20	10	1	91	9	68
2	80	20	10	2	98	2	73
3	80	20	10	3	94	6	75
4	80	20	5	2	100	0	70
5	80	20	2.5	2	100	0	53
6	80	10	5	2	100	0	70
7	80	5	5	2	95	5	66
8	80	2	5	2	95	5	58
9	80	0	5	2	94	6	55
10	50	10	5	2	98	2	70
11	40	10	5	2	98	2	73

a Reaction conditions: 10 g of d-fructose dissolved in DMC/TEAB in the presence of Purolite CT275DR, at 110 °C in an autoclave.

b Estimated via the ^1^H-NMR spectrum.

Considering the previously reported good performances of Amberlyst-15 in the triple dehydration of d-fructose,^14^ we decided to first test its efficiency in autoclave conditions (#1; Table 1) and thus compared the result with other commercially available heterogeneous catalysts (#2–6; Table 1). In all of the performed trials, the autogenous inner pressure never exceeded 2 bar.

After 2 h at 110 °C, the system was cooled down, the reaction mixture was filtered on a celite/basic alumina pad, and a minimal quantity of warm ethyl acetate was used to rinse the autoclave. A dark residue, most probably due to humins and insoluble byproducts, was always present in certain amounts. Nevertheless, both Amberlyst (#1–2; Table 1) and Purolite (#3–6; Table 1) resins showed an almost quantitative selectivity toward HMF, with only a small percentage of OBMF as a byproduct. HMF yield was evaluated to be *ca* 65% in most of the trials (#1–5; Table 1), with the exception of the experiment conducted in the presence of Purolite CT275DR (#6; Table 1), which led to the desired product in 73% yield. This result was ascribed to the high acidity of this Purolite in combination with its large average pore diameter, which might render the acidic sites slightly more accessible (see Table S1, Supporting Information).

The potential participation of DMC in the reaction pathway leading to HMF via methoxycarbonylation (A_Ac_2 mechanism) and/or cyclization reactions (A_Ac_2 + A_Al_2 mechanisms)^23^ was also considered. In fact, recent studies showed that DMC partakes in the esterification/dehydration reactions involved in the conversion of galactaric (mucic) acid to 2,5-furandicarboxylic acid dimethyl ester (FDME).^11^ Thus, in our case study, samples were taken from the best-performing reaction (#6; Table 1) at different time intervals and subjected to ^1^H-NMR analyses (see the Supporting Information). Data collected showed that (i) HMF begins to form already in the first 30 minutes of the reaction; and (ii) there was no evidence of any methoxycarbonylated compounds deriving from the reaction of d-fructose with DMC. These observations strongly suggest that DMC is not involved in the triple dehydration reaction, behaving merely as an extracting solvent.

Once the appropriate catalyst was found, several optimization trials were conducted. For these experiments, the scale of the reaction was increased to 10 grams of d-fructose (Table 2). Initial tests were carried out evaluating the effect of the reaction time (#1–3, Table 2). After 1 hour, HMF selectivity and yield were slightly lower than the ones achieved after 2 h at 110 °C. On the other hand, a prolonged reaction time, i.e., 3 h, led to similar results to the experiment conducted for 2 h.

Trials carried out using lower amounts of Purolite CT275DR i.e., 5% wt., showed an increase in HMF selectivity (#4–5, Table 2). However, when 2.5% wt. catalyst was employed, a significant decrease in HMF yield was noted probably due to the reduced sugar conversion (#5, Table 2).

Further tests demonstrated that the amount of TEAB could be reduced to 10% wt. without affecting HMF selectivity. Additional decrease in the amount of TEAB led to a significant reduction in the HMF yield (#6–9, Table 2). This could be ascribed to the incomplete solubilization of d-fructose in the reaction media, which ultimately led to the formation of humins derived from sugar degradation.

The effect of substrate concentration was also evaluated; employing a lower DMC amount (#10–11, Table 2) gave results comparable to those achieved using a more diluted d-fructose solution (98% HMF selectivity and 72% ^1^H-NMR yield). Therefore, a similar HMF yield can be obtained by halving the DMC amount, thus allowing a more sustainable procedure.

Tests conducted at higher temperatures (up to 150 °C) produced similar or worse results in terms of HMF selectivity and yield: methyl levulinate (ML) and several byproduct peaks started to appear because of side reactions and product degradation (see Table S2, Supporting Information).

### Scale-Up Synthesis of HMF from d-Fructose

**Figure 2:**
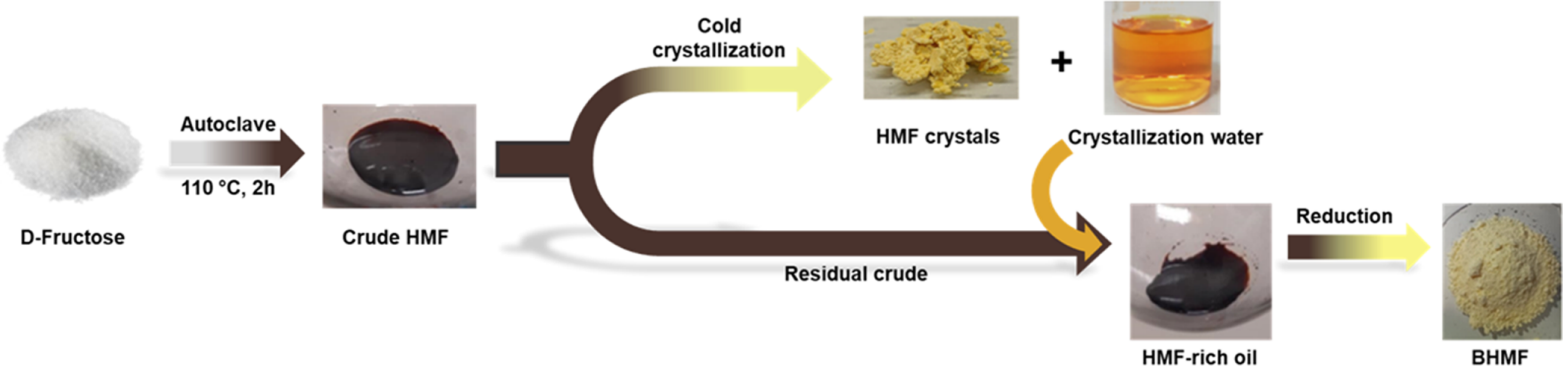
Qualitative representation of the procedure to obtain pure HMF and BHMF.

As mentioned above, most of the research on HMF preparation focuses on finding new synthetic procedures or efficient catalytic systems; however, only limited studies have been reported on developing large-scale synthesis and on purifying the product from the reaction mixture. In consideration of the high HMF market price (from 52 €/g up to 1040 €/g for the analytical standard; prices available on the Sigma-Merk website), attention should be drawn to these issues so as to ultimately lower the HMF cost. In this view, we explored the possibility of using the best-found reaction conditions in the autoclave (#11; Table 2) to test HMF preparation on a larger scale employing up to 40 grams of d-fructose (Table 3) also addressing the purification of the desired product from the reaction mixture (Figure 2) and the waste minimization recovering the excess solvents used.

**Table 3:** Scale-Up HMF Reactions in an Autoclave^a^

			selectivity %^b^						
D-fruct. g	DMC mL	crude HMF g	HMF	OBMF	HMF yield %^b^	HMF crystals yield %	HMF-rich oil g	BHMF yield %^c^	total yield %^d^
10	40	5.37	98	2	73	47	1.86	75 (18)	65
20	80	11.21	98	2	76	44	4.61	78 (21)	65
30	120	17.08	97	2	77	45	6.72	70 (19)	64
40	160	22.76	96	4	72	46	7.92	73 (17)	63

a Reaction conditions: d-fructose was dissolved in DMC/TEAB in the presence of Purolite CT275DR (5% wt.) at 110 °C in an autoclave for 2 h.

b Evaluated via ^1^H-NMR spectroscopy.

c Isolated yield calculated with respect to the HMF contained in the residual oil; in parenthesis, yield calculated with respect to the starting amount of d-fructose.

d Considered as HMF crystals + HMF converted into BHMF.

Results obtained from the scale-up reactions were coherent with those of the 10 gram-scale reaction (Table 2), confirming that our procedure could be a viable pathway to obtain HMF on an even larger scale. In fact, we had to limit our scaling-up trials to 40 grams of d-fructose due to the constraints of the autoclave maximum capacity.

To purify the crude product, a custom-made crystallization process was developed by adopting a procedure available in the literature (Figure 2).^22^ Briefly, after filtration of the reaction mixture on a Gooch packed with celite and basic alumina, the solvents (DMC and EtOAc) were removed under reduced pressure to give a brown oil, which was subsequently dissolved in the solvent selected for the crystallization, i.e., diethyl ether, ethyl acetate, hexane, tetrahydrofuran, 2-methyl tetrahydrofuran, and tertbutyl methyl ether (TBME) (see Table S3 in the Supporting Information). Among them, diethyl ether proved to be the most efficient one; the use of diethyl ether/hexane and ethyl acetate/hexane mixtures also led to reasonable amounts of crystalline HMF.

Despite our best effort, *ca* 30% of the crude oil was insoluble in Et_2_O, and this portion was decanted off; meanwhile, the soluble fraction was left to crystallize in a fridge at −30 °C overnight. The resulting light-yellow crystals were recovered by filtration as pure HMF in *ca* 50% yield (Table 3). The ^1^H-NMR spectrum of the insoluble brown oil showed that this material still mainly contained HMF (see the Supporting Information), most likely entrapped in a gluey humin mixture that rendered it insoluble in Et_2_O. As a proof of concept, the brown oil was added to the residual crystallization solution, and the solvent was evaporated (Figure 2). The crude material was then dissolved in THF and subjected to reduction by adding sodium borohydride (NaBH_4_). As a result, BHMF was isolated as a pale-yellow powder with 70–78% yield—calculated from the amount of brown oil—corresponding to 17–21% yield with respect to the starting amount of d-fructose employed (entries 1–4; Table 3). To have a comprehensive insight into this procedure, it should also be mentioned that a certain mass loss was also evident. As reported in Table 3, HMF and a small amount of OBMF were the only products detected in the reaction mixture with an overall yield of ca 80% (2 mol of HMF is required to achieve 1 mol of OBMF). An insoluble black tar material, most probably humins, was always present at the end of the reaction, which was removed by filtration on celite during the purification procedure.

**Table 4:** Comparison between the Procedure Reported in Our Previous Work and in This Work

							yield %		
	method	DMC mL	TEAB (% wt)	catalyst (% wt)	*T* (°C)	*t* (h)	oil	crystals	reaction scale (g)^a^
prev. work^14^	reflux	80	20	Amb-15 (10)	90	16	70		20
this work	autoclave	40	10	CT275DR (5)	110	2	73	47	40

a Maximum amount of d-fructose used.

Table 4 reports a comparison between our previously reported procedure for HMF synthesis^14^ and the one herein presented. Although both procedures are based on a TEAB/DMC biphasic system and on using commercially available reagents, catalysts, and solvents, in this improved procedure, Purolite CT275DR proved to be the best catalyst in promoting the triple dehydration and the reaction time was drastically reduced from 16 to 2 h. It was also possible to decrease the amount of TEAB and DMC without affecting the reaction outcome. Both DMC and the ethyl acetate used during the workup can be recovered and reused (see the Green Metrics Analysis section). Furthermore, the custom-made purification procedure for the isolation of HMF and BHMF—herein proposed for the first time—was also developed following the principles of waste minimization and solvent reuse.

It should be finally mentioned that for industrial applications another viable purification procedure could be distillation as the HMF boiling point was reported to be 112–114 °C at 1 mmHg.

### Green Metrics Analysis in HMF Syntheses

Green metrics are good tools to evaluate reaction performances: AE (atom economy), PMI (process mass intensity), RME (reaction mass efficiency), MRP (material recovery parameter), and *E*-factor are only some of the well-established metrics that can help in the investigation of different reaction aspects.^24^

Briefly, AE measures the fraction of atoms found in the reagents that end up in the product structure, calculated as the ratio of the molecular weight of the product to the sum of the molecular weights of reagents.^25^ PMI represents the mass (kg) of all input materials (reagents, catalysts, and solvents) employed to produce 1 kg of the target product(s).^25,26^ RME is expressed as the ratio of the mass of the target product collected to the sum of the masses of all input materials multiplied by 100%; effectively, it is the inverse of PMI. MRP is related to the amount of workup solvents and materials used during the synthetic process that could be potentially recovered by recycling processes.^25^ The *E*-factor (or *E*-total) calculates the kilograms of waste produced per kilogram of product. In addition, the *E*-factor can be fractioned into several contributions, which investigate a specific type of waste: E-kernel, E-reaction solvent (E-rnx solv), E-catalyst (E-cat), E-workup, and E-purification (E-purif).^25,26^ Further insights into the aforementioned metrics are given in the Supporting Information.

Since HMF isolation is a key step to making this compound available for further functionalization reactions as well as to render the process exploitable at an industrial scale, having an idea of the efficiency of the synthetic process is of paramount importance. For this reason, we decided to compare the herein-proposed new procedure with the other selected synthetic approaches reported in the literature that isolate HMF through crystallization, distillation, or column chromatography, starting from at least 0.5 g of d-fructose (Figure 2, Supporting Information, Table S4 and Figure S1).

Entries marked with a star refer to articles that were already evaluated in our previous work^14^ and that we have decided to include also in this study for completeness. For each procedure, the radial pentagon method^27^ was also employed; this visual tool quantifies the performances of each synthetic procedure with respect to AE, PMI, RME, MRP, and *E*-f (see the Supporting Information).

It must be mentioned that not all of the experimental procedures reported the specific amounts of the employed materials, particularly those related to the purification procedures. For this reason, the calculated green metrics may, in some cases, lack accuracy and should therefore be regarded as minimum estimates with respect to PMI and *E*-factor quantities.

**Figure 3:**
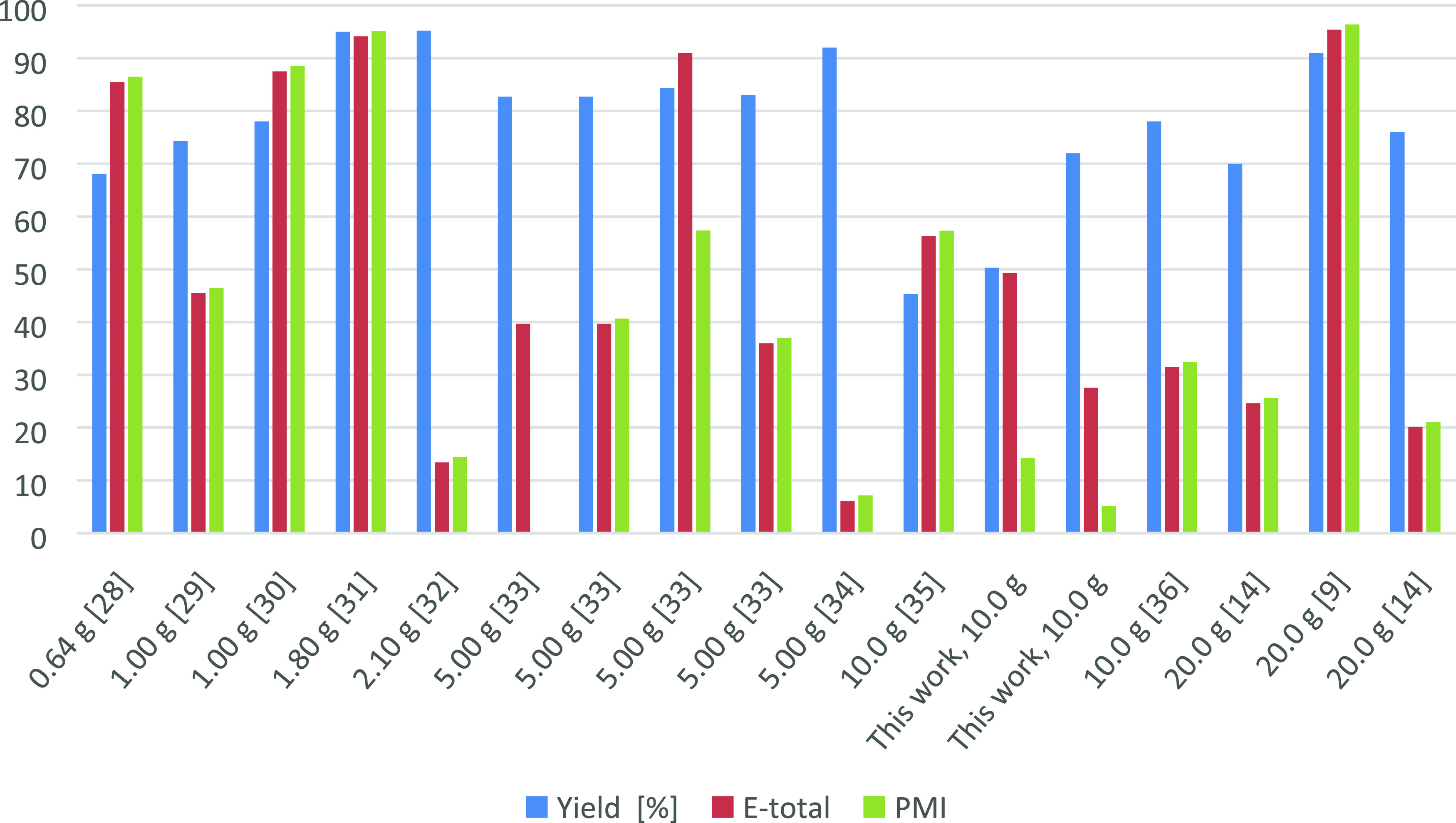
Comparison of yield (%), *E*-total, and PMI values of synthetic routes to HMF having PMI < 100, with the scale of the reaction given below each set of bars.

The method and the amount of d-fructose, catalysts, and solvents greatly differ in each procedure (see Table S4) and ultimately lead to very heterogeneous results. For clarity, we decided to classify the collected data into two groups: reactions with PMI lower than 100 (#1–17, Table S4 and Figure 3) and reactions with PMI higher than 100 (#18–24, Table S4 and Figure S1 Supporting Information).

From these premises, the histogram reported in Figure 3 compares the yield, *E*-factor, and PMI of the procedures that had the lowest PMI values (<100); data are presented considering the amount of d-fructose used, which increases from the left to the right.

The key features that identify a sustainable/green procedure are high yield and low values of *E*-factor and PMI; indeed, they reflect a consistent formation of the product with limited waste production. According to the data analyzed, the overall HMF yields range from moderate (45.3%)^35−39^ to almost quantitative (95.2%),^32^ independently from the amount of starting material employed.

Focusing on the waste-related metrics (*E*-factor and PMI), it is evident that the Dibenedetto,^28^ Tong,^30^ Rajmohan,^31^ and Simeonov^9^ procedures display a consistent waste production (between 80 and 100 Kg for each Kg of HMF), rendering these procedures not particularly effective from an environmental point of view. Both *E*-factor and PMI values are highly influenced by the amount of solvents used for HMF isolation and purification.

The procedure reported in this work, together with those developed by Novamont^32^ and Motokucho et al.,^34^ presents a lower value of waste-related metrics (PMI < 20) while maintaining high HMF yields. It is also important to note that the increase in the reaction scale generally leads to a decrease in the amount of waste produced, with a slight reduction in reaction yield.

According to these data, the best-performing procedure seems to be the one developed by Motokucho et al.^34^ due to its almost quantitative yield, very low PMI, and the lowest *E*-factor among the screened works. Nevertheless, this reaction requires heating at 90 °C in an autoclave for 168 h, under CO_2_ pressure (7.0 MPa); moreover, the isolation of pure HMF employs column chromatography with chloroform as the eluent. Once again, this observation highlights the importance of carefully evaluating the green metrics within the context of the methodology used as, for instance, reaction time, toxicity of the compounds, and temperature are, in fact, not considered by the green metrics. Despite the high PMI value, the procedure developed by Galkin and co-workers^22^ is one of the few focusing on HMF synthesis and isolation in the large scale as well as the first example of its successful crystallization.

Compared to the other works, our procedure provides many advantages as it encompasses (i) cheap and nontoxic reagents; (ii) short reaction time and low catalyst loading; (iii) easy scalable approach; and (iv) isolation of high-purity crystalline HMF and BHMF. Another important aspect of this improved methodology is that the extracting solvent (DMC) and the workup solvent (ethyl acetate) can be easily recovered and reused. As an example, in a typical 10 gram-scale procedure, the reaction mixture from the autoclave was filtered on paper into a round-bottom flask; then, DMC was distilled with a rotatory evaporator, allowing for the recovery of 88% of the total amount used (35 mL). The residual viscous mixture, the autoclave, and the paper filter were washed with EtOAc; the organic fraction was filtered on a Gooch packed with basic alumina and celite and collected into a flask. Again, distillation of the organic solvent led to the recovery of 75% (90 mL) EtOAc. This reflects directly in the PMI value, which is one of the lowest among all of the reported procedures (PMI = 5.13).

### Ecoscale Evaluation of HMF Synthetic Approaches

Another useful semiquantitative tool to compare the greenness of reactions is Ecoscale, an algorithm that assesses not only quantities but also material toxicity and hazard, costs, reaction setup, time, temperature, workup, and purification of a synthetic process.^40^ From an initial score of 100, the software removes points considering the following attributes: the presence of dangerous reagents and wastes, consuming workup procedures, the use of sophisticated equipment, and of course the reaction yield; the higher the final score, the better the reaction.

**Figure 4:**
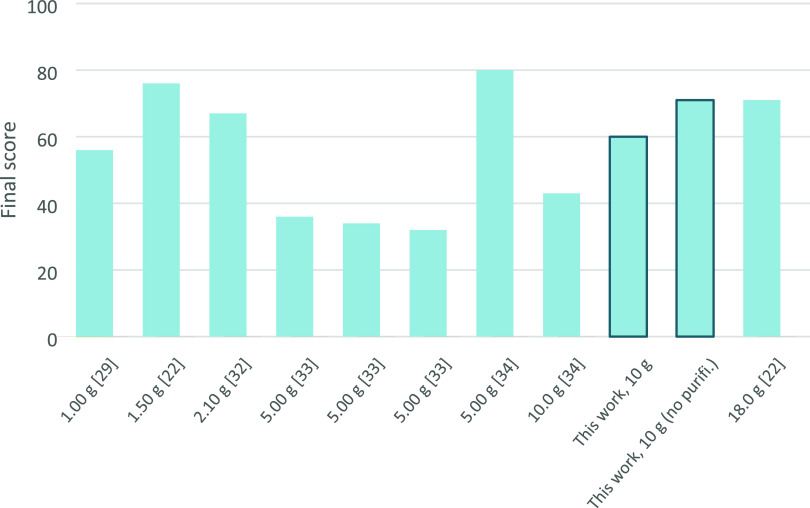
Ecoscale values for the best-performing HMF synthetic procedures (see Table S5 in the Supporting Information), with the scale of the reaction given below each set of bars.

Figure 4 reports the scores of the procedures listed in Table S4 (#2, 5, 6, 8–13, 18, 22, Table S4; see the Supporting Information) selected among the best-performing HMF syntheses. Since Ecoscale recognizes substances through their CAS number, some reagents and/or custom-made catalysts could not be considered in the evaluation. To build a more realistic comparison, substances employed for the synthesis of catalysts were also added in the reagent section of the algorithm. However, it must be pointed out that, generally, catalysts are recovered and reused several times, which is not considered by the program. On this basis, the herein-proposed procedure reached a maximum final score of 71 (60 when considering purification), which is among the highest scores, and it is comparable once again to the results of Galkin et al.^22^ and Novamont’s patent.^32^ Shi et al.^33^ achieved very low scores, despite having a high yield mainly due to the two-step catalyst synthesis. The suggested best procedure is the one of Motokucho et al.,^34^ which couples high yield and nonharmful reagents; however, as mentioned above, it required 7 days of heating in an autoclave and purification employing a halogenated solvent.

The evaluation of the Brasholz et al.^29^ resulted in a medium score, while the method of Kovash et al.^35^ was negatively influenced mainly by the hazard and toxicity of reagents.

## Conclusions

In this work, we report an efficient, fast, green, and easily scalable method for the synthesis and isolation of HMF (ca 50% yield) and BHMF (ca 20% yield) starting from d-fructose (up to 40 grams). The procedure employs inexpensive reagents, catalysts, and solvents, and the desired product, HMF, can be isolated via minimal purification as a yellow crystalline compound. The residual HMF-rich oil insoluble in diethyl ether, used as a solvent for crystallization, was easily reduced to BHMF, which was also recovered as a pure compound.

To have a complete evaluation of our procedure, a comparison with selected HMF syntheses reported in the literature was carried out employing several green metrics, a visual radial pentagon analysis, and Ecoscale. Data collected using these different tools led to similar findings, i.e., they revealed the greenness of the synthetic approach herein proposed, especially considering the good PMI values, which remained relatively low even when considering the HMF purification procedure. Regarding HMF yield and selectivity, our synthesis was among the best reported so far in the literature, although this comparison is limited to procedures reporting isolated yields. Despite these good results, it is evident that additional studies must be conducted especially to achieve *E*-factor ≤5, which is the common target value of the refinery industry.

## Experimental Section

### General

All of the reagents and solvents were purchased from Sigma-Aldrich and employed without any further purification. Purolite CT275DR was kindly provided by Purolite. TEAB was dried in an oven at 100 °C overnight before use. Reactions were conducted in a stainless-steel autoclave (capacity 220 mL) with a thermocouple for temperature control and under magnetic stirring (1000 rpm). NMR spectra were acquired through a spectrometer Bruker 400 MHz in CDCl_3_ and MeOD.

### Synthesis of HMF in an Autoclave (#11, Table 2)

In a typical reaction in an autoclave, 10.0 g of d-fructose (0.055 mol, 1 mol. equiv) was reacted with 1.0 g of TEAB (10% wt.), 0.5 g of Purolite CT275DR (5% wt.), and 40 mL of dimethyl carbonate (0.475 mol, 8.6 mol. equiv), at 110 °C for 2 h. The autogenous pressure reached the value of 2 bar. After cooling, the reaction crude was filtered on a Gooch under vacuum with basic alumina (5 g) and celite (5 g) and washed with hot ethyl acetate (30 mL × 4). The mixture was then evaporated and dried under vacuum to give a viscous dark-brown oil (5.30 g). HMF yield (73%) in the crude product was estimated by ^1^H-NMR. ^1^H-NMR (400 MHz; CDCl_3_) δ (ppm): 9.64 (s, 1H), 7.24 (d, 1H), 6.54 (d, 1H) and 4.75 (s, 2H). ^13^C-NMR (100 MHz; CDCl_3_) δ (ppm): 177.64, 160.47, 152.43, 122.61, 109.99, 57.68.

### HMF Crystallization

The HMF-containing reaction crude was dissolved in 30 mL of Et_2_O (10 mL × 3). The organic yellow layer was separated from the insoluble dark-brown oil, and both were stored at −30 °C for 48 h. Orange-yellow crystals of HMF were filtered on a paper filter and dried under a vacuum to give pure HMF (3.17 g, 47%). The filtered mixture and the insoluble dark-brown oil obtained in the previous step were mixed together and dried under a vacuum.

### Synthesis of HMF in an Autoclave: Large Scale (#4, Table 3)

In a typical reaction in an autoclave, 40.0 g of d-fructose (0.222 mol, 1 mol. equiv) was reacted with 4.0 g of TEAB (10% wt.), 2.0 g of Purolite CT275DR (5% wt.), and 160 mL of dimethyl carbonate (1.93 mol, 8.6 mol. equiv), at 110 °C for 2 h. The autogenous pressure reached the value of 8 bar. After cooling, the reaction crude was filtered on a Gooch under vacuum with basic alumina (15 g) and celite (15 g) and washed with hot ethyl acetate (80 mL × 5). The mixture was then evaporated and dried under a vacuum to give the crude product as a viscous dark-brown oil (7.92 g). HMF yield (72%) in the crude product was estimated by ^1^H-NMR.

### HMF Crystallization: Large Scale

The HMF-containing reaction crude was dissolved in 90 mL of Et_2_O (30 mL × 3). The organic yellow layer was separated from the insoluble dark-brown oil, and both were stored at −30 °C for 48 h. Orange-yellow crystals of HMF were filtered on a paper filter and dried under a vacuum to give pure HMF (12.94 g, 46%). The filtered Et_2_O and the insoluble dark-brown oil obtained in the previous step were mixed together and dried under a vacuum.

### Synthesis of 5-Bis(hydroxymethyl)furan (BHMF)

The dark-brown HMF mixture obtained in previous steps (4.61 g) was dissolved in 110 mL of THF; then, 2.08 g (1.5 mol. equiv) of NaBH_4_ was added slowly under stirring. The mixture was allowed to react overnight at room temperature. The mixture was quenched by the addition of water (20 mL), and the organic solvent was concentrated under a vacuum. The aqueous mixture was transferred in a separatory funnel and extracted with 30 mL × 3 of AcOEt. The organic fractions were collected and dried with Na_2_SO_4_, filtered, and concentrated under a vacuum to give 2.98 g of pure BHMF as a yellow solid (21% with respect to the initial 20 g of d-fructose). The product was further purified by grinding it in a mortar and pestle in the presence of 15 mL of Et_2_O; the solvent was then removed with a Pasteur pipette, obtaining a pale-yellow powder. ^1^H-NMR (400 MHz; MeOD) δ (ppm): 6.25 (s, 2H), 4.51 (s, 4H). ^13^C-NMR (100 MHz; MeOD) δ (ppm): 154.36, 107.71, 56.08.

## Supporting Information Available

Catalyst properties’ overview, ^1^H-NMR spectra analyses of a typical HMF synthesis at different reaction times; some additional experimental details and purification methods; green metrics evaluation tables, including Ecoscale evaluation, *E*-factor, PMI, and visual radial pentagon analysis; and ^1^H-NMR spectra for all compounds isolated (PDF)

## References

[ref1] aZhao Y.; Fu Y.; Guo Q. X. Production of aromatic hydrocarbons through catalytic pyrolysis of γ-valerolactone from biomass. Bioresour. Technol. 2012, 114, 740–744. 10.1016/j.biortech.2012.03.057. 22507905

[ref2] Xu C.; Paone E.; Rodríguez-Padrón D.; Luque R.; Mauriello M. Recent catalytic routes for the preparation and the upgrading of biomass derived furfural and 5-hydroxymethylfurfural. Chem. Soc. Rev. 2020, 49, 4273–4306. 10.1039/D0CS00041H. 32453311

[ref3] Xia H.; Xu S.; Hu H.; An J.; Li C. Efficient conversion of 5-hydroxymethylfurfural to high-value chemicals by chemo- and bio-catalysis. RSC Adv. 2018, 8, 30875–30886. 10.1039/C8RA05308A. 35548764 PMC9085621

[ref4] Yang Z.-Z.; Deng J.; Pan T.; Guo Q.; Fu Y. A one-pot approach for conversion of fructose to 2, 5-diformylfuran by combination of Fe_3_O_4_-SBA-SO_3_H and K-OMS-2. Green Chem. 2012, 14, 2986–2989. 10.1039/c2gc35947b.

[ref5] Zakrzewska M. E.; Bogel-łukasik E.; Bogel-łukasik R. Ionic Liquid-Mediated Formation of 5-Hydroxymethylfurfural—A Promising Biomass-Derived Building Block. Chem Rev. 2011, 111, 397–417. 10.1021/cr100171a. 20973468

[ref6] Román-Leshkov Y.; Barrett C. J.; Liu Z. Y.; Dumesic J. A. Production of dimethylfuran for liquid fuels from biomass-derived carbohydrates. Nature 2007, 447, 982–986. 10.1038/nature05923. 17581580

[ref7] Jing S.; Cao X.; Zhong L.; Peng X.; Sun R.; Liu J. Effectively enhancing conversion of cellulose to HMF by combining in-situ carbonic acid from CO_2_ and metal oxides. Ind. Crops. Prod. 2018, 126, 151–157. 10.1016/j.indcrop.2018.10.028.

[ref8] Krystof M.; Pérez-Sánchez M.; De María P. D. Lipase-catalyzed (Trans) esterification of 5-hydroxy-methylfurfural and separation from HMF esters using deep-eutectic solvents. ChemSusChem. 2013, 6, 630–634. 10.1002/cssc.201200931. 23456887

[ref9] Simeonov S. P.; Coelho J. A. S.; Afonso C. A. M. An Integrated Approach for the Production and Isolation of 5-Hydroxymethylfurfural from Carbohydrates. ChemSusChem. 2012, 5, 1388–1391. 10.1002/cssc.201200236. 22740298

[ref10] Hou Q.; Zhen M.; Liu L.; Chen Y.; Huang F.; Zhang S.; Li W.; Ju M. Tin phosphate as a heterogeneous catalyst for efficient dehydration of glucose into 5-hydroxymethylfurfural in ionic liquid. Appl. Catal., B. 2018, 224, 183–193. 10.1016/j.apcatb.2017.09.049.

[ref11] Trapasso G.; Annatelli M.; Dalla Torre D.; Aricò F. Synthesis of 2, 5-furandicarboxylic acid dimethyl ester from galactaric acid via dimethyl carbonate chemistry. Green Chem. 2022, 24, 2766–2771. 10.1039/D1GC04408G.

[ref12] aAricò F. Synthetic approaches to 2, 5-bis (hydroxymethyl) furan (BHMF): a stable bio-based diol. Pure Appl. Chem. 2021, 93, 551–560. 10.1515/pac-2021-0117.

[ref13] Averochkin G. M.; Gordeev E. G.; Skorobogatko M. K.; Kucherov F. A.; Ananikov V. P. Systematic Study of Aromatic-Ring-Targeted Cycloadditions of 5-Hydroxymethylfurfural Platform Chemicals. ChemSusChem. 2021, 14, 3110–3123. 10.1002/cssc.202100818. 34060725

[ref14] Musolino M.; Andraos J.; Aricò F. An easy scalable approach to HMF employing DMC as reaction media: reaction optimization and comparative environmental assessment. ChemistrySelect 2018, 3, 2359–2365. 10.1002/slct.201800198.

[ref15] Esmaeili N.; Zohuriaan-Mehr M. J.; Bouhendi H.; Bagheri-Marandi G. HMF synthesis in aqueous and organic media under ultrasonication, microwave irradiation and conventional heating. Korean J. Chem. Eng. 2016, 33, 1964–1970. 10.1007/s11814-016-0031-8.

[ref16] Zhang T.; Hu Y.; Huai L.; Gao Z.; Zhang J. Facile synthesis and isolation of 5-hydroxymethylfurfural from diphenyl sulfoxide. Green Chem. 2021, 23, 3241–3245. 10.1039/D1GC00302J.

[ref17] Hansen T. S.; Woodley J. M.; Riisager A. Efficient microwave-assisted synthesis of 5-hydroxymethylfurfural from concentrated aqueous fructose. Carbohydr. Res. 2009, 344, 2568–2572. 10.1016/j.carres.2009.09.036. 19850284

[ref18] Christian T. J.; Manley-harris M.; Field R. J.; Parker B. A. Kinetics of Formation of Di-d-fructose Dianhydrides during Thermal Treatment of Inulin. J. Agric. Food Chem. 2000, 48, 1823–1837. 10.1021/jf9911186. 10820101

[ref19] Van Dam H. E.; Kieboom A. P. G.; Van Bekkum H. The conversion of fructose and glucose in acidic media: formation of hydroxymethylfurfural. Starch-Stärke 1986, 38, 95–101. 10.1002/star.19860380308.

[ref20] Rosenfeld C.; Konnerth J.; Sailer-Kronlachner W.; Solt P.; Rosenau T.; Van Herwijnen H. W. G. Current situation of the challenging scale-up development of hydroxymethylfurfural production. ChemSusChem. 2020, 13, 3544–3564. 10.1002/cssc.202000581. 32302054 PMC7496312

[ref21] Rosatella A. A.; Simeonov S. P.; Frate R.F.M.; Alfonso C. A. M. 5-Hydroxymethylfurfural (HMF) as a building block platform: Biological properties, synthesis and synthetic applications. Green Chem. 2011, 13, 754–793. 10.1039/c0gc00401d.

[ref22] Galkin K. I.; Krivodaeva E. A.; Romashov L. V.; Zalesskiy S. S.; Kachala V. V.; Burykina J. V.; Ananikov V. P. Critical Influence of 5-Hydroxymethylfurfural Aging and Decomposition on the Utility of Biomass Conversion in Organic Synthesis. Angew. Chem., Int. Ed. 2016, 55, 8338–8342. 10.1002/anie.201602883. 27271823

[ref23] Tundo P.; Musolino M.; Aricò F. The reactions of dimethyl carbonate and its derivatives. Green Chem. 2018, 20, 28–85. 10.1039/C7GC01764B.

[ref24] Andraos J.; Hent A. Simplified Application of Material Efficiency Green Metrics to Synthesis Plans: Pedagogical Case Studies Selected from Organic Syntheses. J. Chem.Educ. 2015, 92, 1820–1830. 10.1021/acs.jchemed.5b00058.

[ref25] Andraos J. Green Chemistry Metrics: Material Efficiency and Strategic Synthesis Design. In Innovations in Green Chemistry and Green Engineering; Anastas P.; Zimmerman J., Eds.; Springer: New York, NY, 2013.

[ref26] Jimenez-Gonzalez C.; Ponder C. S.; Broxterman Q. B.; Manley J. B. Using the right green yardstick: why process mass intensity is used in the pharmaceutical industry to drive more sustainable processes. Org. Process Res. Dev. 2011, 15, 912–917. 10.1021/op200097d.

[ref27] Andraos J.; Sayed M. On the use of″ green″ metrics in the undergraduate organic chemistry lecture and lab to assess the mass efficiency of organic reactions. J. Chem. Educ. 2007, 84, 1004–1010. 10.1021/ed084p1004.

[ref28] Dibenedetto A.; Aresta M.; Pastore C.; di Bitonto L.; Angelini A.; Quaranta E. Conversion of fructose into 5-HMF: a study on the behaviour of heterogeneous cerium-based catalysts and their stability in aqueous media under mild conditions. RSC Adv. 2015, 5, 26941–26948. 10.1039/C5RA03358F.

[ref29] Brasholz M.; von Kanel K.; Hornung C. H.; Saubern S.; Tsanaktsidis J. Highly efficient dehydration of carbohydrates to 5-(chloromethyl)furfural (CMF), 5-(hydroxymethyl)furfural (HMF) and levulinic acid by biphasic continuous flow processing. Green Chem. 2011, 13, 1114–1117. 10.1039/c1gc15107j.

[ref30] Tong X.; Li M.; Yan N.; Ma Y.; Dyson P. J.; Li Y. Defunctionalization of fructose and sucrose: Iron-catalyzed production of 5-hydroxymethylfurfural from fructose and sucrose. Catal. Today. 2011, 175, 524–527. 10.1016/j.cattod.2011.03.003.

[ref31] Rajmohan R.; Gayathri S.; Vairaprakash P. Facile synthesis of 5-hydroxymethylfurfural: a sustainable raw material for the synthesis of key intermediates toward 21, 23-dioxaporphyrins. RSC Adv. 2015, 5, 100401–100407. 10.1039/C5RA19400H.

[ref32] Capuzzi L.; Digioia F.; Carotenuto G. Process for the synthesis of 5-hydroxymethylfurfural from saccharides. WO2014/180979A12014.

[ref33] Shi X.-L.; Zhang M.; Li Y.; Zhang W. Polypropylene fiber supported ionic liquids for the conversion of fructose to 5-hydroxymethylfurfural under mild conditions. Green Chem. 2013, 15, 3438–3445. 10.1039/c3gc41565a.

[ref34] Motokucho S.; Moriwara H.; Nakatani H.; Noordover B. A. J. Efficient and environmental-friendly dehydration of fructose to 5-hydroxymethyl-2-furfural in water under high pressure of CO_2_. Tetrahedron Lett. 2016, 57, 4742–4745. 10.1016/j.tetlet.2016.09.036.

[ref35] Kovash C. S.; Pavlacky E.; Selvakumar S.; Sibi M. P.; Webster D. C. Thermoset coatings from epoxidized sucrose soyate and blocked, bio-based dicarboxylic acids. ChemSusChem 2014, 7, 2289–2294. 10.1002/cssc.201402091. 24777954

[ref36] Brown D. W.; Floyd A. J.; Kinsman R. G.; Roshan-Ali Y. Dehydration reactions of fructose in non-aqueous media. J. Chem. Technol. Biotechnol. 1982, 32, 920–924. 10.1002/jctb.5030320730.

[ref37] Vinke P.; Van Bekkum H. The dehydration of fructose towards 5-hydroxymethylfurfural using activated carbon as adsorbent. Starch 1992, 44, 90–96. 10.1002/star.19920440303.

[ref38] Musau R. M.; Munavu R. M. The preparation of 5-hydroxymethyl-2-furaldehyde (HMF) from d-fructose in the presence of DMSO. Biomass 1987, 13, 67–74. 10.1016/0144-4565(87)90072-2.

[ref39] Chan J. Y. G.; Zhang Y. Selective conversion of fructose to 5-hydroxymethylfurfural catalyzed by tungsten salts at low temperatures. ChemSusChem. 2009, 2, 731–734. 10.1002/cssc.200900117. 19650105

[ref40] aVan Aken K.; Strekowski L.; Patiny L. EcoScale, a semi-quantitative tool to select an organic preparation based on economical and ecological parameters Beilstein J. Org. Chem. 2006, 2 (3), 10.1186/1860-5397-2-3. PMC1409775 16542013

