# Supplementary material for: Multigram Synthesis of Pure HMF and BHMF
Source: Org Process Res Dev. 2022 Sep 9;26(10):2830–8. doi: 10.1021/acs.oprd.2c00196 (PMC9594346; doi:10.1021/acs.oprd.2c00196)

Supporting Information

A multi-gram synthesis to pure HMF and BHMF

Giacomo Trapasso, Giovanna Mazzi, Beatriz Chícharo, Mattia Annatelli, Davide Dalla Torre, Fabio Aricò

Dr. G. Trapasso, Dr. M. Annatelli, G. Mazzi, Dr. B. Chícharo, Dr. D. Dalla Torre, Prof. F. Aricò;

Department of Environmental Sciences Informatics and Statistics, Ca' Foscari University of Venice, Via Torino, 155, 30170 Mestre, Venezia (IT).

Contents

Catalysts properties overview 2

^{1}H-NMR spectra of a typical HMF synthesis at different times 3

(samples taken from #6 Table 1 reaction) 3

Additional reactions for the synthesis of HMF in autoclave 4

^{1}H-NMR of #3, Table S2 in $CDCl_3$. 4

Crystallization/Purification of HMF 5

Green metrics evaluation 6

Explanation of waste-related Green Metrics: 6

Comprehensive table for the evaluation of green metrics 7

Ecoscale evaluation........8

Comparison of Yield, E-factor and PMI of reactions with PMI > 100. 9

Radical pentagon analysis........9

^{1}NMR and ^{13}C-NMR spectra 14

Catalysts properties overview

Table S1. Purolites and Amberlysts properties.

	Amberlyst-15	Amberlyst-36	CT151	CT269	CT275
Polymer structure	Macroporous polystyrene crosslinked with divinylbenzene				
Appearance	Spherical Beads				
Functional Group	Sulfonic Acid				
Ionic Form	H^+ form				
Particle Size Range	< 300 µm	600-850 µm	425 - 1200 µm		
Dry Weight Capacity	≥ 4.7 eq/kg (H^+ form)	≥ 5.4 eq/kg (H^+ form)	5.1 eq/kg (H^+ form)	5.2 eq/kg (H^+ form)	5.2 eq/kg (H^+ form)
Moisture Retention	≤ 1.6% (H^+ form)	51 - 57 % (H^+ form)	54 - 59 % (H^+ form)	51 - 57 % (H^+ form)	51 - 59 % (H^+ form)
Surface Area	53 m^2/g	33 m^2/g	15 - 25 m^2/g	35 - 50 m^2/g	20 - 40 m^2/g
Pore Volume	0.40 mL/g	0.20 mL/g	0.15 - 0.30 mL/g	0.30 - 0.50 mL/g	0.40 - 0.60 mL/g
Average Pore Diameter	300 Å	240 Å	250 - 400 Å	250 - 425 Å	400 - 700 Å
Temperature Limit	120 °C	150 °C	150 °C	130 °C	130 °C

a) All Purolite® information is available on https://www.purolite.com/index. All Amberlyst-15 information are available on the DuPont (https://www.dupont.com/content/dam/dupont/amer/us/en/watersolutions/public/documents/en/45-D00927-en.pdf).

^{1}H-NMR spectra of a typical HMF synthesis at different times

(samples taken from #6 Table 1 reaction)

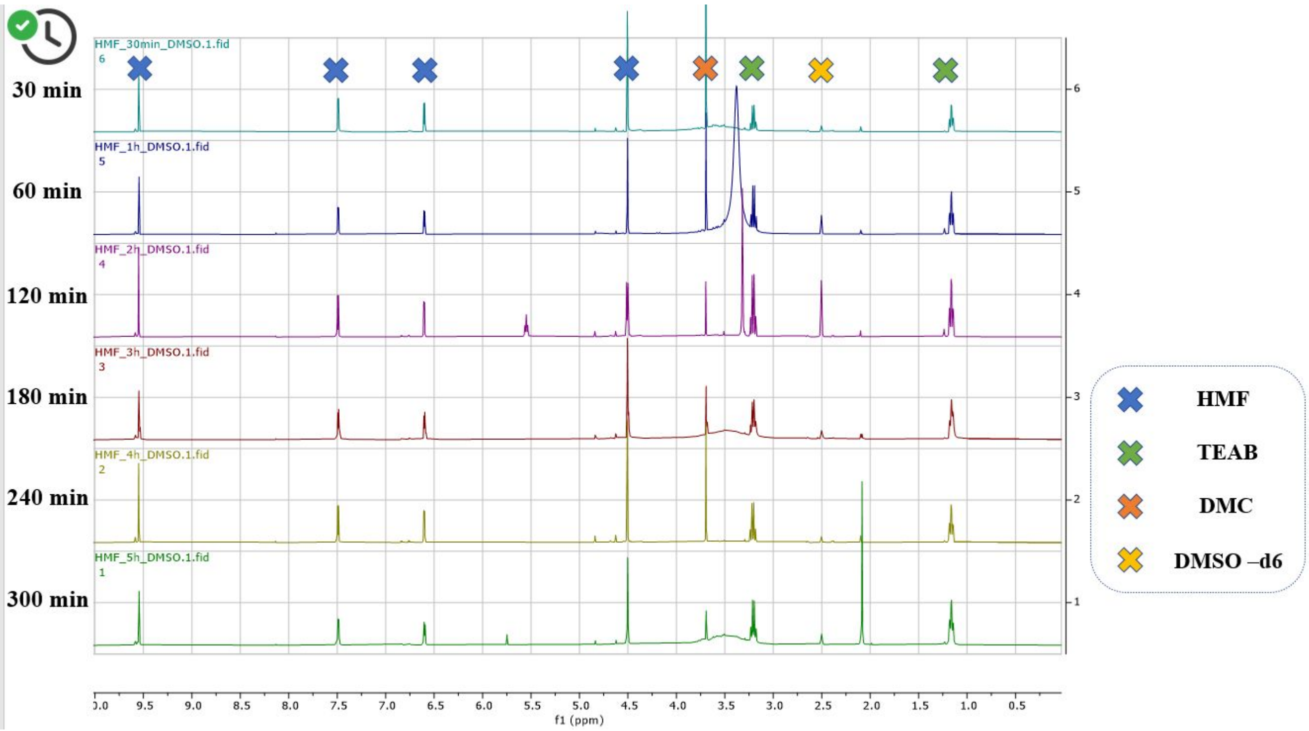

Additional reactions for the synthesis of HMF in autoclave

Table S2. *Synthesis of HMF in autoclave with temperatures higher than 110 °C.*

#	DMC [mL]	TEAB [%]	Purolite CT275DR [%]	T [C°]	Time [h]	^{1}H-NMR Selectivity [%] HMF	ML	Other	HMF ^{1}H-NMR Yield [%]
**1**	40	10% wt.	5% wt.	120	2	**97**	3	0	68
**2**	40	10% wt.	5% wt.	130	2	**96**	4	0	70
**3**	40	10% wt.	5% wt.	150	2	**68**	18	16	46
**4**	40	10% wt.	5% wt.	150	4	**71**	4	25	60

^{1}H-NMR of #3, Table S2 in $CDCl_3$.

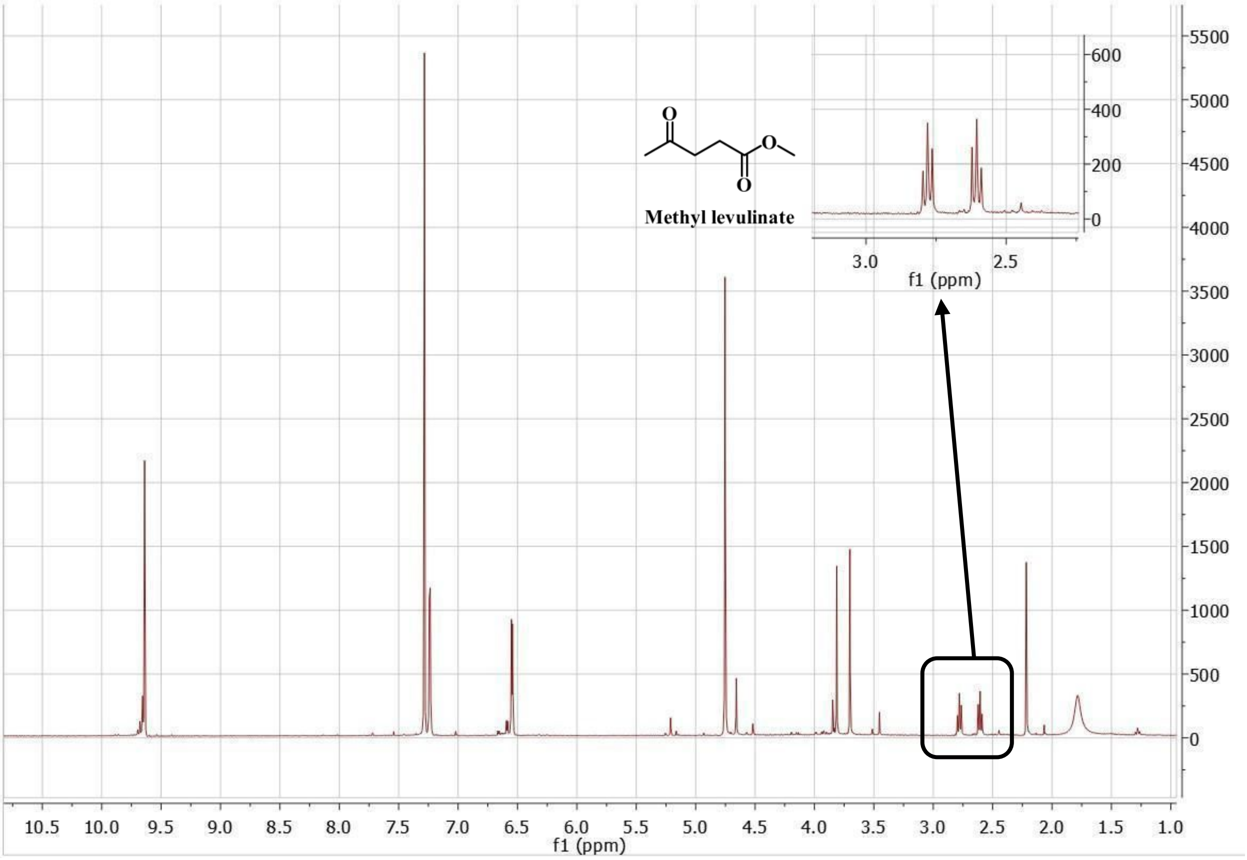

Crystallization/Purification of HMF

Table S3. Solvents used in the HMF crystallization trials.[a]

#	Solvent	HMF crystals	Crystal yield %	Crystals
1[b]	Et_2O	✓	ca. 50	Bubble-shape, yellow crystals 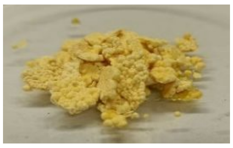
2[b,d]	Et_2O + Hexane	✓	ca. 30	Needle-shape, yellow-orange crystals. 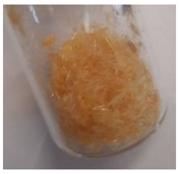
3[c]	t-butyl methyl ether (TBME)	✗	/	/
4[c]	THF	✗	/	/
5[c]	2-MeTHF	✗	/	/
6[c]	Hexane	✗	/	/
7[b]	Acetone + Hexane	✗	/	/
8[b,e]	AcOEt + Hexane	✓	ca. 30	Needle-shape, yellow-orange. They melt faster. 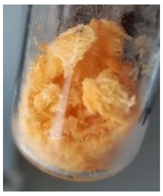
9[c]	AcOEt	✗	/	/

[a] After obtaining the dark brown reaction crude, solvent(s) was added, the organic phase was collected in a beaker and put in freezer for 48h; [b] Separation between organic phase and dark brown crude oil; [c] Solvent dissolves the dark brown crude oil; [d] 10 mL of Et_2O were poured in the reaction crude, than hexane was added until the formation of a white-yellow powder; this procedure repeated two more times, the recovered organic phase were then put in the freezer for 48h; [e] Dark brown oil dissolved in 5 mL of AcOEt, than hexane was added until the formation of a white-yellow powder; this procedure repeated two more times, the recovered organic phase were then put in the freezer for 48h.

Green metrics evaluation

Explanation of waste-related Green Metrics:

- E-kernel: Mass contribution to the total E-factor from reaction by-products, reaction side products, and unreacted starting materials;
- E-reaction solvent (E-rxn solv): Mass of reaction solvent necessary for the synthesis of the target product;
- E-catalyst (E-cat): Mass of the catalyst necessary for the synthesis of the target product;
- E-workup: Mass of the reagents used in the work-up procedures necessary to obtain the target product;
- E-purification (E-purif): Mass of the reagents used in purification procedures necessary to obtain the pure target product;

Comprehensive table for the evaluation of green metrics

Table S4. *Environmental assessment of different procedures with PMI lower than 100 (#1-17) and higher than 100 (#18-25).*

#	Method[b]	D-Fructose [g]	Catalyst	Rxn solvent	Yield [%]	E-kernel	E- rxn solv.	E-cat	E-workup	E-purif	E-total	PMI	Ref.
1*	B	0.64	CeP_3[c]	DMC-water	68	1.11	44.43	0.33	39.6	0	85.47	86.47	Dibenedetto *et al.*[28]
2[d]	C	1	HCl 0.25M	MIBK	74.3	0.92	23.02	21.54	0	0	45.48	46.48	Brasholz *et al.*[29]
3*	B	1	$FeCl_3/Et_4NBr$	NMP[e]	78	0.83	18.83	0.55	67.29	0	87.5	88.5	Tong *et al.*[30]
4*[f]	B	1.8	HBr/silica	THF	95	0.5	88.8	4.01	0.83	>>0	94.14	95.14	Rajmohan *et al.*[31]
5[d]	B	2.1	Ti/Si500	Dist water/TEAC	95.2	0.5	7.14	0.14	5.64	0	13.42	14.42	Novamont[32]
6[g]	B	5	$[PPFPy][HSO_4]$	DMSO	82.7	0.73	30.4	0.92	7.6	0	39.65	4.17[h]	Shi *et al.*[33]
7	B	5	$[PPFPy][HSO_4]$	DMSO	82.7	0.73	30.4	0.92	7.6	0	39.65	40.65	Shi *et al.*[33]
8[g]	B	5	$[PPFPy][HSO_4]$	DMSO	84.4	0.69	29.79	0.9	59.58	0	90.97	57.34[h]	Shi *et al.*[33]
9	B	5	$[PPFPy][HSO_4]$	MIBK/water	83	0.72	28.86	0.92	5.49	0	35.99	36.99	Shi *et al.*[33]
10[f],[i]	A	5	CO_2	H_2O	92	0.55	5.59	0	0	0	6.14	7.14	Motokucho *et al.*[34]
11[d],[f]	B	10	H_2SO_4/LiBr	DMAc	45.3	2.15	29.56	3.26	21.34	0	56.31	57.31	Kovash *et al.*[35]
12[g]	A	10	Pur CT275DR	DMC	50.3	2.03	12.96	0.45	27.3	6.49	49.24	14.25	This work
13	A	10	Pur CT275DR	DMC	72	0.98	8.45	0.3	17.8	0	27.53	5.13	This work
14*[f]	B	10	Amb-15	CH_3CN:TEAC	78	0.82	21.27	2.18	7.19	0	31.46	32.46	Brown *et al.*[36]
15*	B	20	Amb-15	DMC:TEAB	70	1.04	17.45	0.61	5.52	0	24.62	25.62	Musolino *et al.*[14]
16*	B	20	Amb-15	water	91	0.57	0.7	7.28	86.84	0	95.39	96.39	Simeonov *et al.*[9]
17*	B	20	BF_3 OEt_2	DMC	76	0.89	16.14	0.53	2.55	0	20.11	21.11	Musolino *et al.*[14]
18[m],[g]	B	1.5	H_2SO_4	[BMIM][Cl]	78	0.83	8.55	0.02	605.74	34.68	649.81	650.81	Galkin *et al.*[22]
19[m]	B	1.5	H_2SO_4	[BMIM][Cl]	78	0.83	8.55	0.02	605.74	0	615.13	616.13	Galkin *et al.*[22]
20*	B	9	Cationic resin/activate carbon	water	48	1.97	49.5	19.8	260.4	0	331.67	332.67	Vinke *et al.*[37]
21*	B	18	BF_3 OEt_2	DMSO-toluene	60	1.37	26.41	0.29	67.74	153.79	249.60	250.60	Musau *et al.*[38]
22[m],[g]	B	18	H_2SO_4	[BMIM][Cl]	72.9	0.96	7.83	0.02	647.79	37.03	693.69	694.69	Galkin *et al.*[22]
23[m]	B	18	H_2SO_4	[BMIM][Cl]	72.9	0.96	7.83	0.02	647.79	0	656.61	657,61	Galkin *et al.*[22]
24*[n]	C	70	WCl_6/HY	[BMIM]Cl/THF	55	1.62	170.04	0.27	0	0	171.93	172.93	Chan *et al.*[39]

[a] E-excess is not reported in the table as its value was zero for all the synthetic procedure reported; the reported metrics do not consider the preparation of the catalyst; procedures signed by a star were already reported in our previous article [13] [b] A = Autoclave, B = Batch, C = Continuous flow; [c] CeP_3 is Cerium Phosphate catalyst with formula $[(Ce(PO_4)1.5(H_2O)(H_3O)0.5(H_2O)_{0.5})]$; [d] Amounts of work-up and/or purification materials are not reported in the original article; [e] NMP = N-Methyl-2-pyrrolidone; [f] Excluding column chromatography; [g] Including purification; [h] DMSO is partially recovered; [i] Reaction conducted for 148 h; [l] NHC is 1,3-bis(2,6-diisopropylbenzyl)imidazolylidene; [m] Aqueous solution of NaCl/NaHCO3 not included; [n] Reaction conducted in continuous biphasic system over a 42 h period where 7 cycles of 10 g each of D-fructose run at 6 h intervals were combined.

Ecoscale evaluation

Table S5. Ecoscale scores for the selected procedures for HMF synthesis and isolation.

Reference	Amount of D-fructose g	Ecoscale value	Comments
Brasholz *et al.*[29]	1.00	56	Continuous flow reaction
Galkin *et al.*[22]	1.5	76	Purification included
Novamont[32]	2.10	89	Ti/Si 500 (catalyst) not included
		67	Reagent for catalyst synthesis included
Shi *et al.*[33]	5.00	83	[PPFPy][HSO_4] not included, DMSO recovered by distillation from reaction crude
		36	Reagent for catalyst synthesis included
Shi *et al.*[33]	5.00	81	[PPFPy][HSO_4] not included, DMSO recovered by distillation from reaction crude; purification by AcOEt/H_2O extraction
		34	Reagent for catalyst synthesis included
Shi *et al.*[33]	5.00	82	MIBK/water extraction
		32	Reagent for catalyst synthesis included
Motokucho *et al.*[34]	5.00	80	Reaction performed for 168 h in autoclave; amount of CO_2 employed not specified
Kovash *et al.*[35]	10	43	HMF isolated as yellow liquid
This work with purif. (50 % yield)	10	60	Purolite not available in the database (Amb-15 used instead of Purolite)
This work, no purif. (72% yield)	10	71	Purolite not available in the database (Amb-15 used instead of purolite)
Galkin *et al.*[22]	18	71	Large-scale reaction in rotatory evaporator

Comparison of Yield, E-factor and PMI of reactions with PMI > 100.

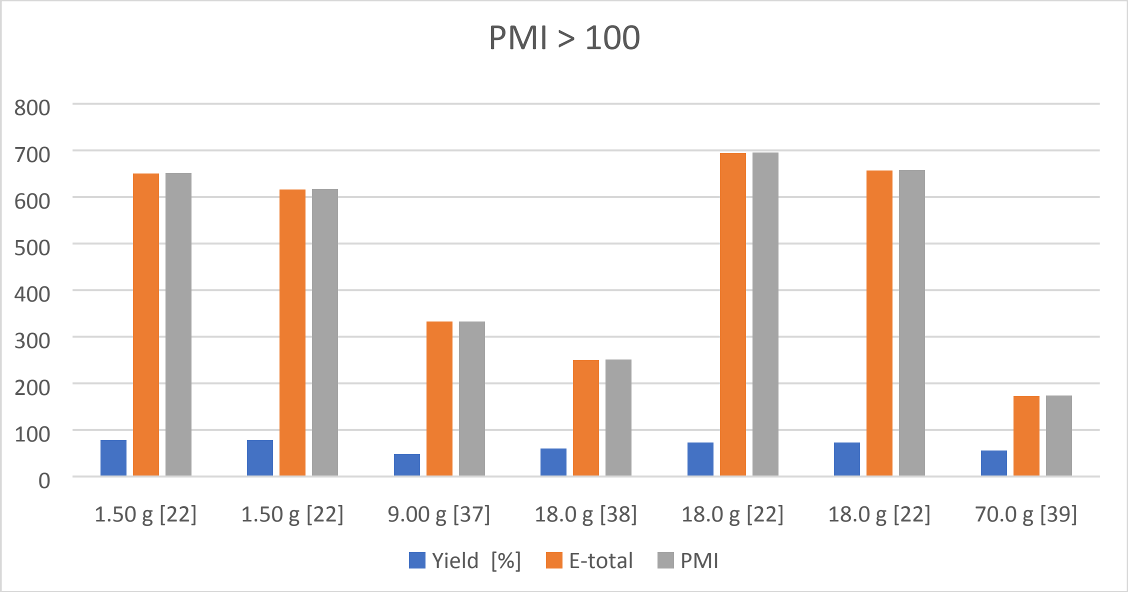

Figure S1. *Comparison of yield %, E-total and Process Max Intensity (PMI) of procedures with PMI higher than 100; with the amount of D-Fructose employed below each set of bars.*

Radical pentagon analysis

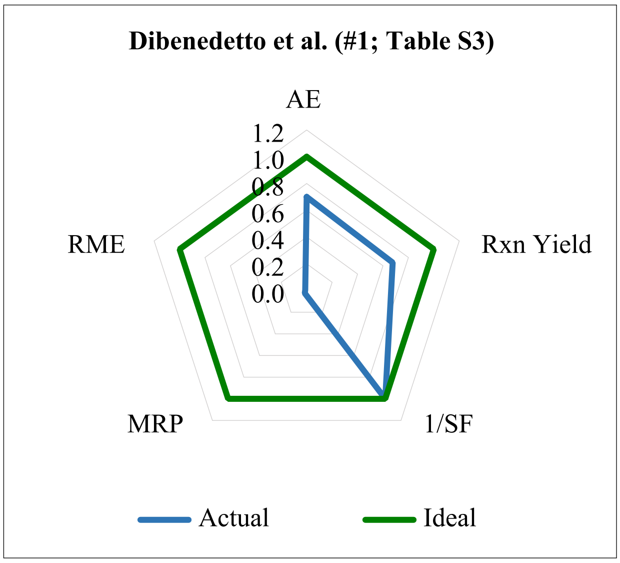

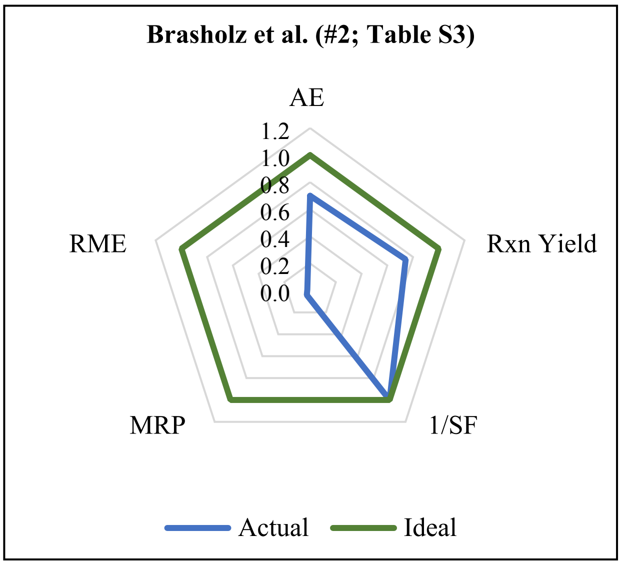

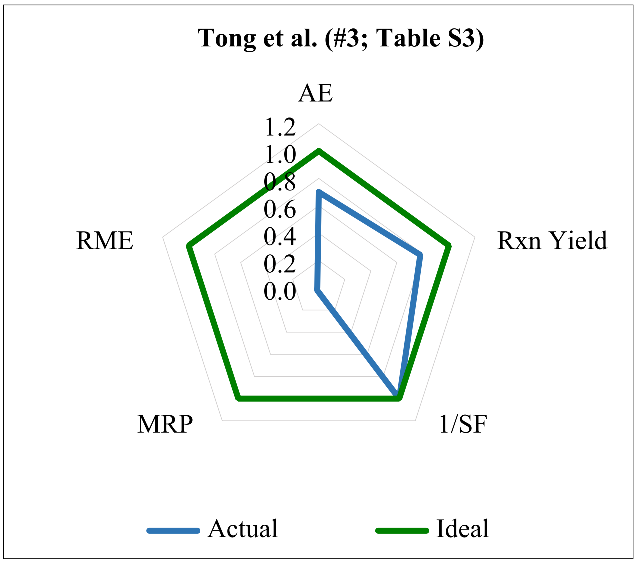
Tong et al. (#3; Table S3)
AE
1.2
1.0
0.8
0.6
0.4
0.2
0.0
Rxn Yield
1/SF
MRP
RME
Actual
Ideal

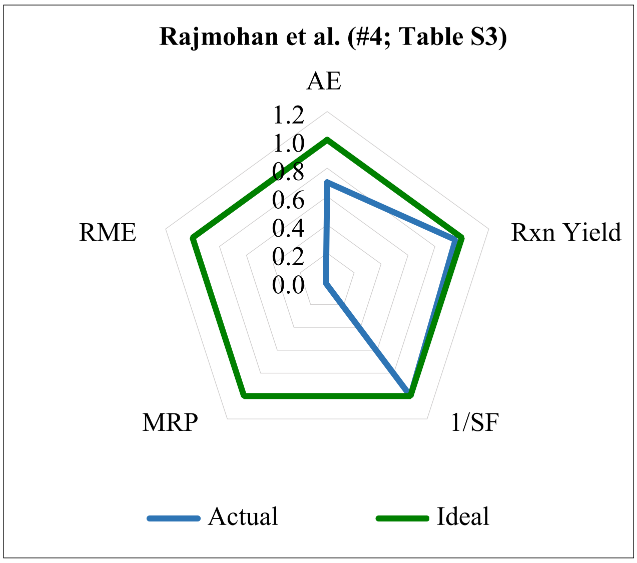
Rajmohan et al. (#4; Table S3)
AE
1.2
1.0
0.8
0.6
0.4
0.2
0.0
Rxn Yield
1/SF
MRP
RME
Actual
Ideal

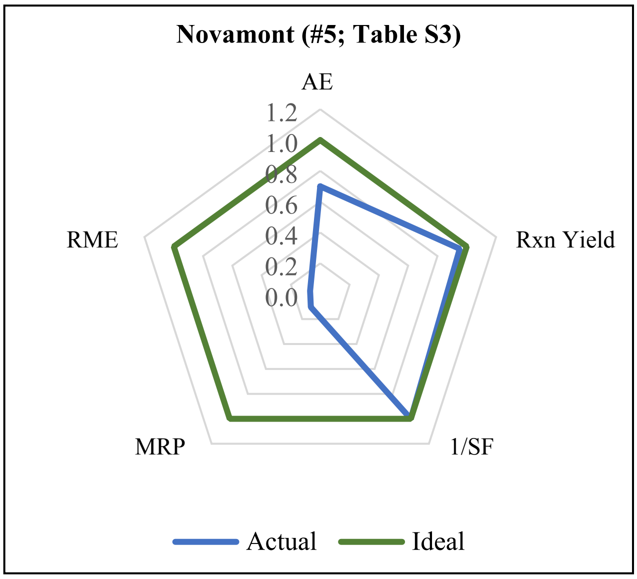
Novamont (#5; Table S3)
AE
1.2
1.0
0.8
0.6
0.4
0.2
0.0
Rxn Yield
1/SF
MRP
RME
Actual
Ideal

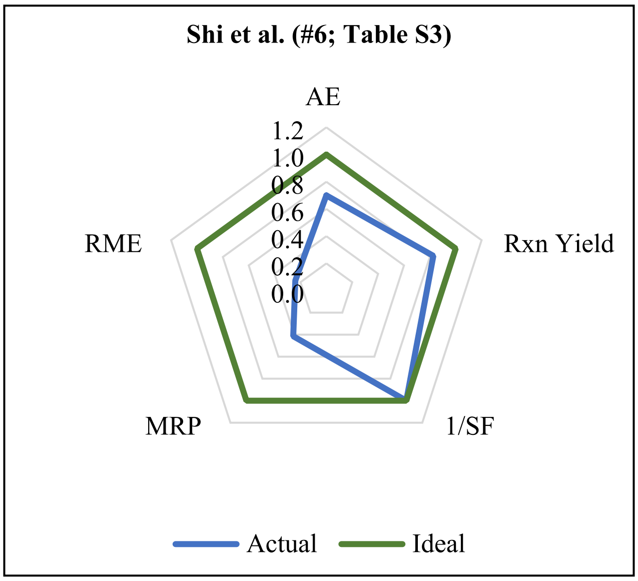
Shi et al. (#6; Table S3)
AE
1.2
1.0
0.8
0.6
0.4
0.2
0.0
Rxn Yield
1/SF
MRP
RME
Actual
Ideal

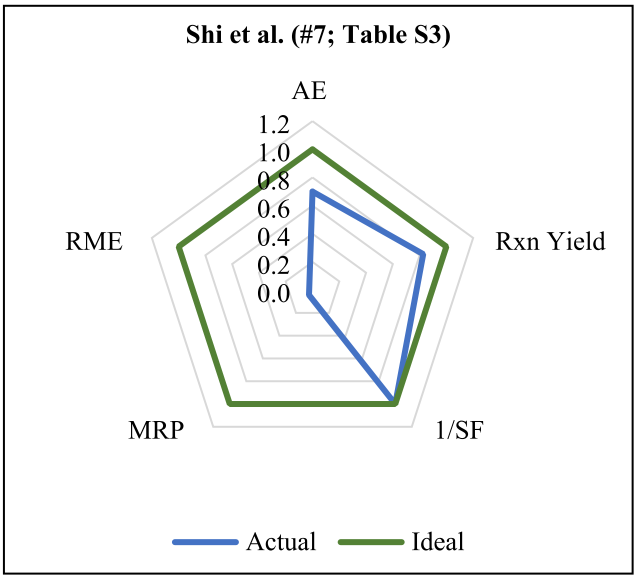
Shi et al. (#7; Table S3)
AE
1.2
1.0
0.8
0.6
0.4
0.2
0.0
Rxn Yield
1/SF
MRP
RME
Actual
Ideal

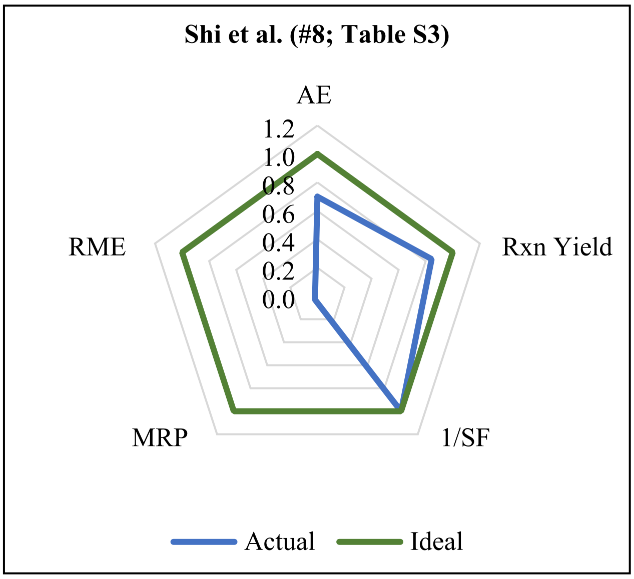
Shi et al. (#8; Table S3)
AE
1.2
1.0
0.8
0.6
0.4
0.2
0.0
Rxn Yield
1/SF
MRP
RME
Actual
Ideal

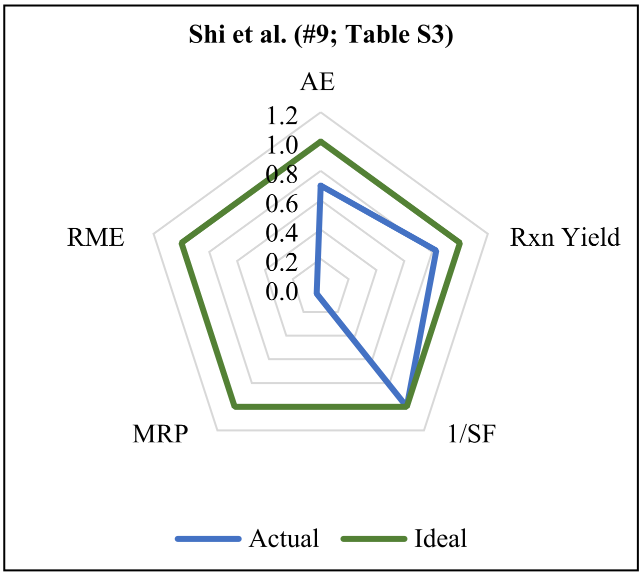
Shi et al. (#9; Table S3)
AE
1.2
1.0
0.8
0.6
0.4
0.2
0.0
Rxn Yield
1/SF
MRP
RME
Actual
Ideal

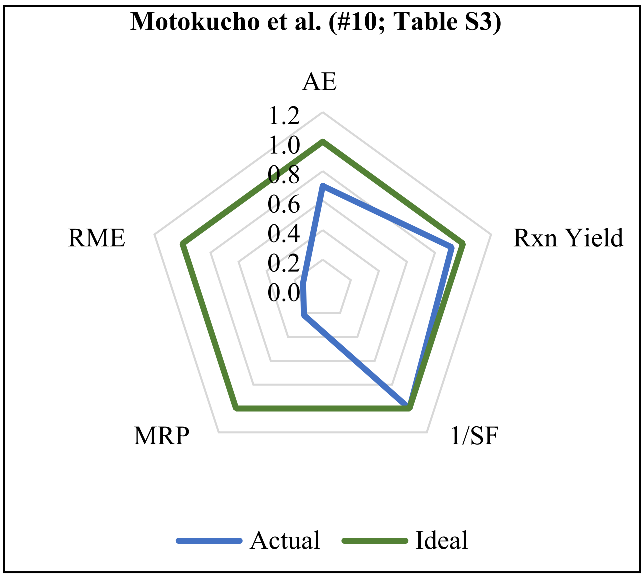
Motokucho et al. (#10; Table S3)
AE
1.2
1.0
0.8
0.6
0.4
0.2
0.0
Rxn Yield
1/SF
MRP
RME
Actual
Ideal

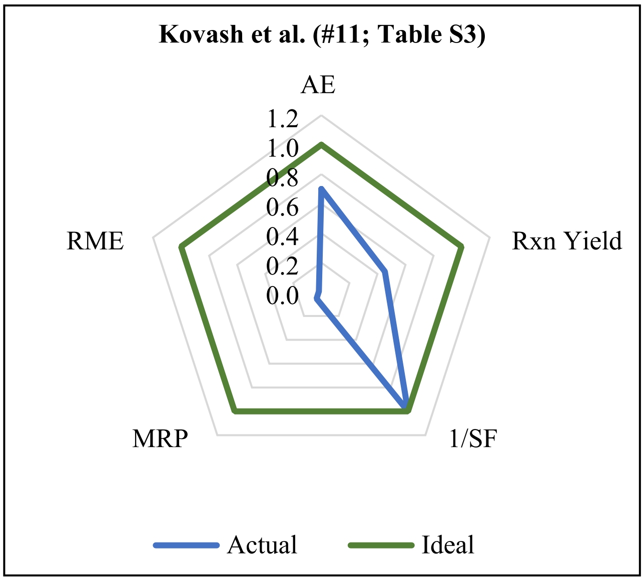
Kovash et al. (#11; Table S3)
AE
1.2
1.0
0.8
0.6
0.4
0.2
0.0
Rxn Yield
1/SF
MRP
RME
Actual
Ideal

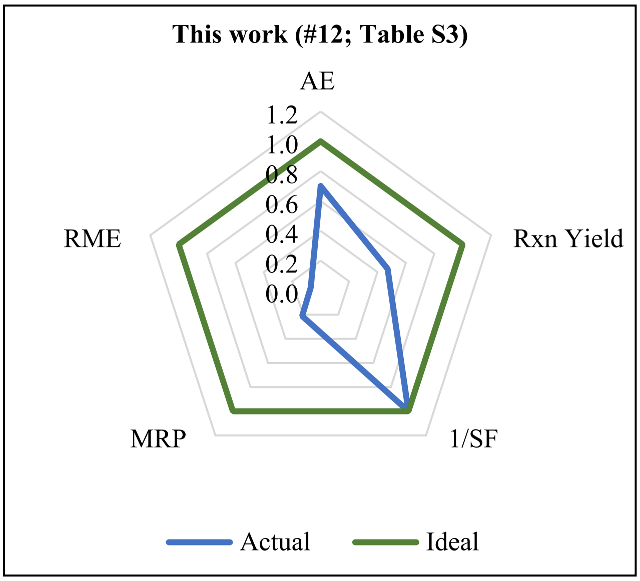
This work (#12; Table S3)
AE
1.2
1.0
0.8
0.6
0.4
0.2
0.0
Rxn Yield
1/SF
MRP
RME
Actual
Ideal

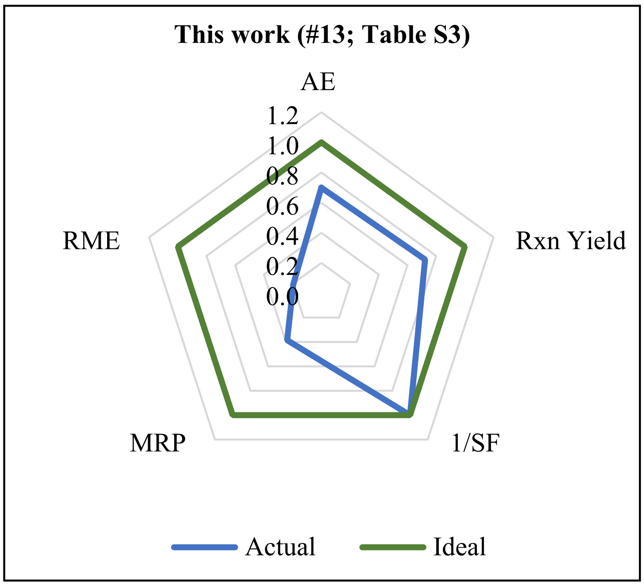
This work (#13; Table S3)
AE
1.2
1.0
0.8
0.6
0.4
0.2
0.0
Rxn Yield
1/SF
MRP
RME
Actual
Ideal

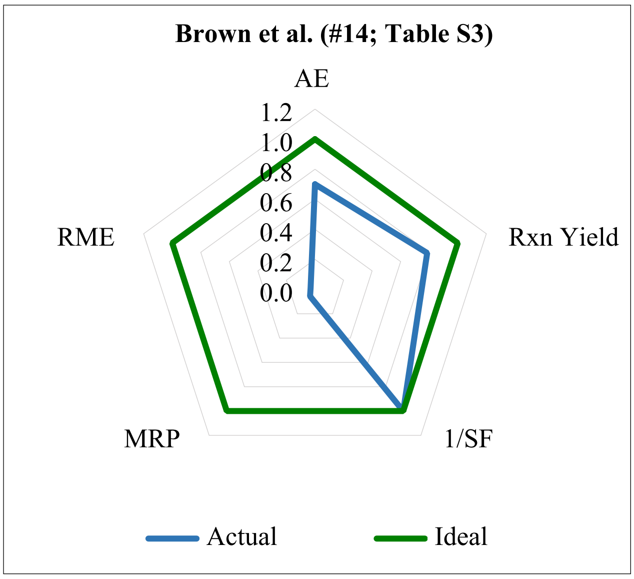
Brown et al. (#14; Table S3)
AE
1.2
1.0
0.8
0.6
0.4
0.2
0.0
Rxn Yield
1/SF
MRP
RME
Actual
Ideal

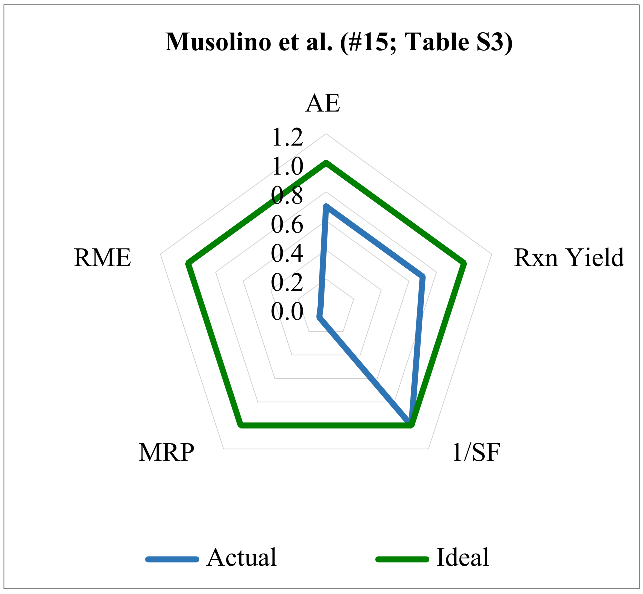
Musolino et al. (#15; Table S3)
AE
Rxn Yield
1/SF
MRP
RME
1.2
1.0
0.8
0.6
0.4
0.2
0.0
Actual
Ideal

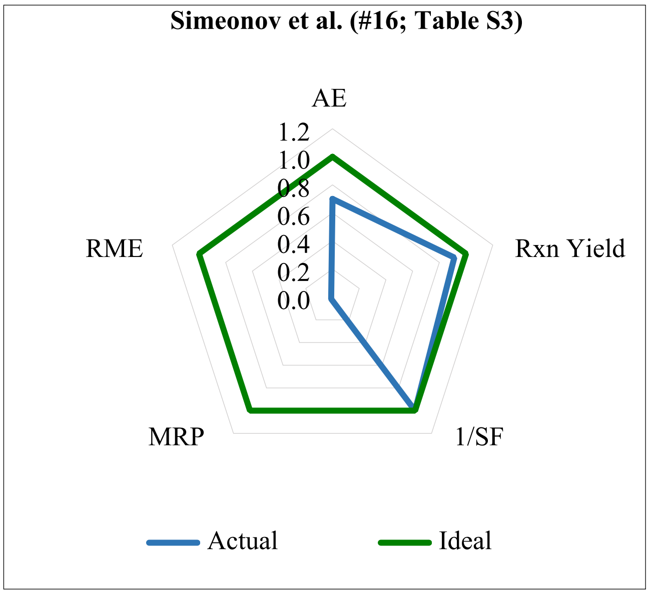
Simeonov et al. (#16; Table S3)
AE
Rxn Yield
1/SF
MRP
RME
1.2
1.0
0.8
0.6
0.4
0.2
0.0
Actual
Ideal

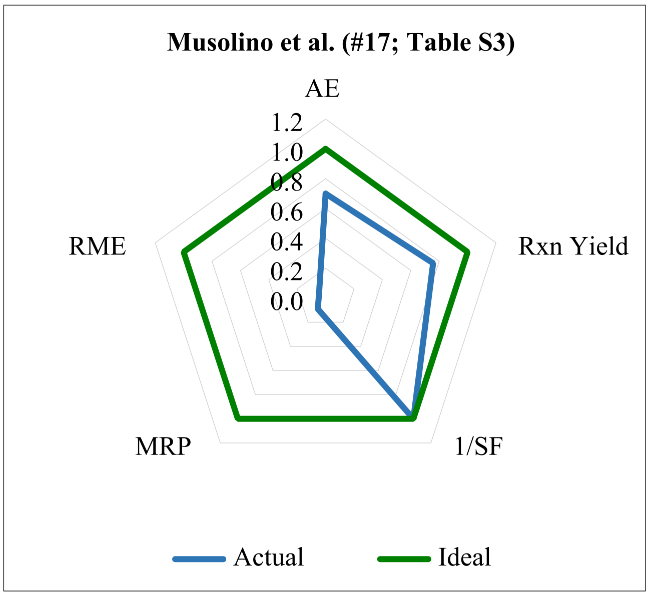
Musolino et al. (#17; Table S3)
AE
Rxn Yield
1/SF
MRP
RME
1.2
1.0
0.8
0.6
0.4
0.2
0.0
Actual
Ideal

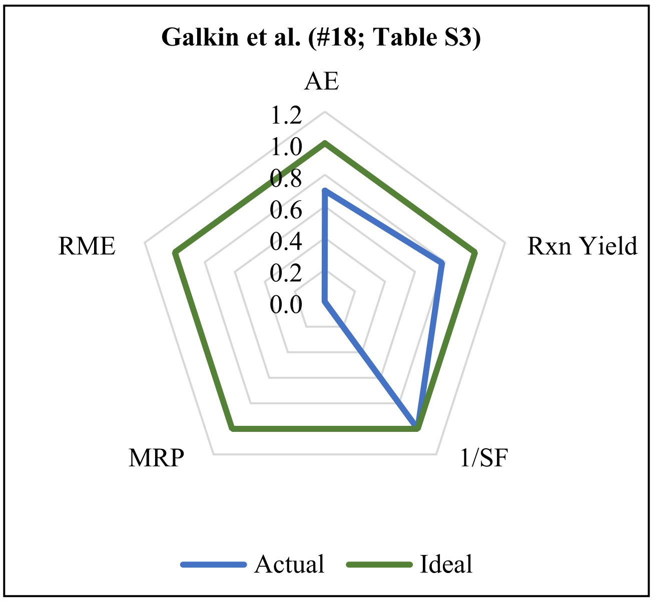
Galkin et al. (#18; Table S3)
AE
Rxn Yield
1/SF
MRP
RME
1.2
1.0
0.8
0.6
0.4
0.2
0.0
Actual
Ideal

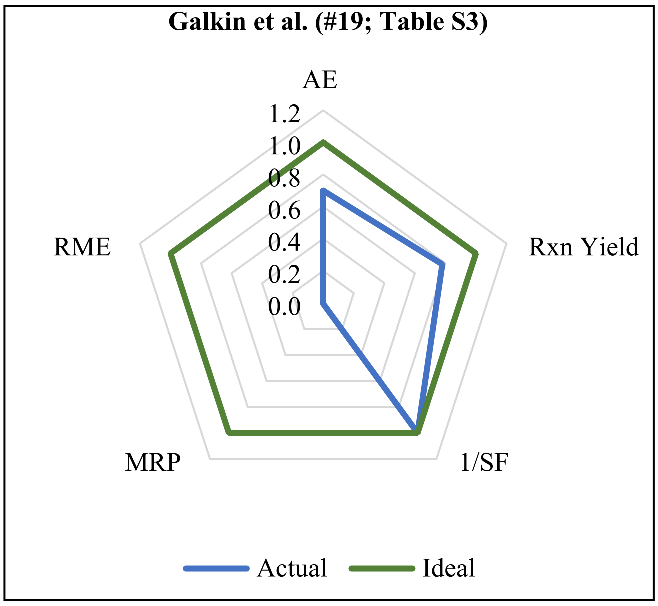
Galkin et al. (#19; Table S3)
AE
Rxn Yield
1/SF
MRP
RME
1.2
1.0
0.8
0.6
0.4
0.2
0.0
Actual
Ideal

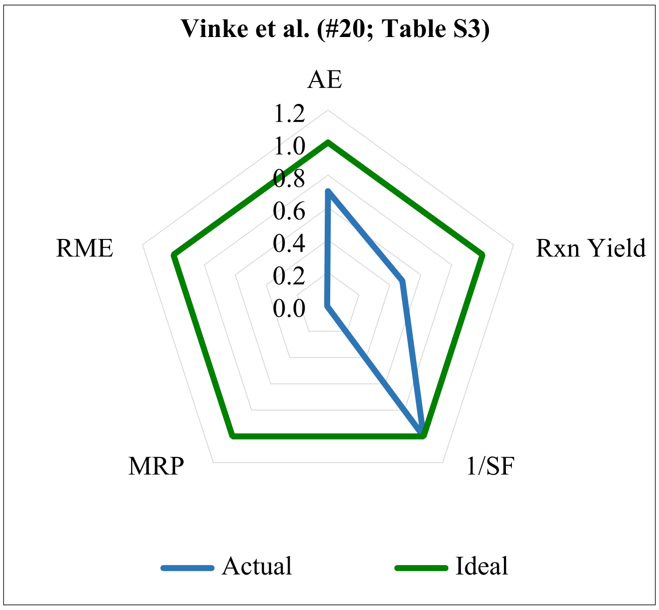
Vinke et al. (#20; Table S3)
AE
Rxn Yield
1/SF
MRP
RME
1.2
1.0
0.8
0.6
0.4
0.2
0.0
Actual
Ideal

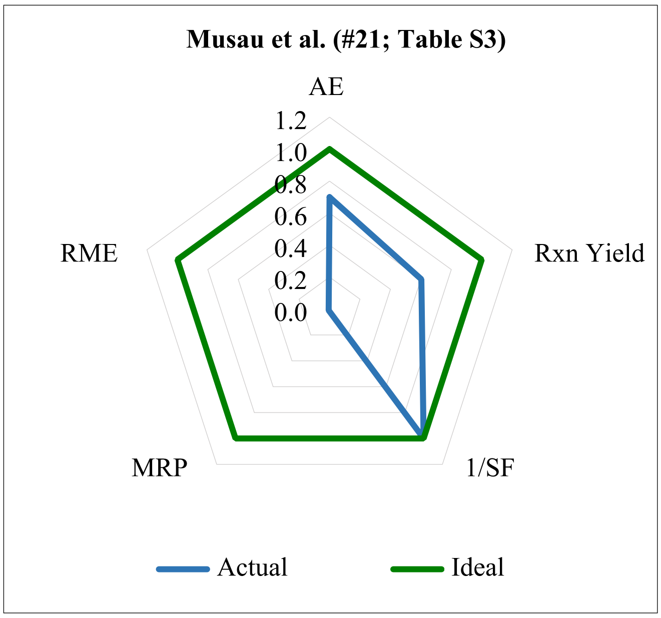

Musau et al. (#21; Table S3)
AE
1.2
1.0
0.8
0.6
0.4
0.2
0.0
Rxn Yield
1/SF
MRP
RME
Actual
Ideal

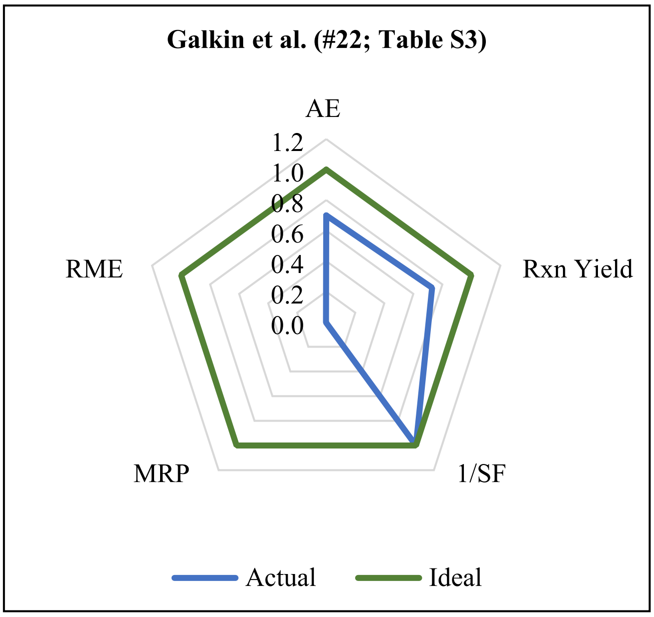

Galkin et al. (#22; Table S3)
AE
1.2
1.0
0.8
0.6
0.4
0.2
0.0
Rxn Yield
1/SF
MRP
RME
Actual
Ideal

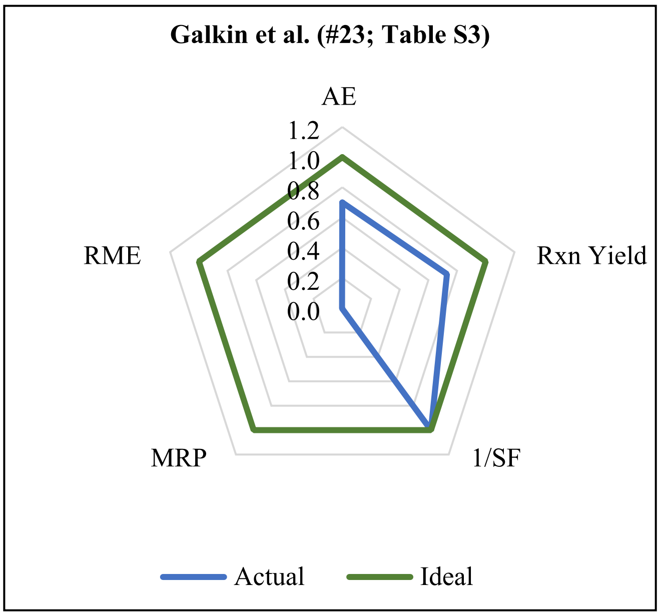

Galkin et al. (#23; Table S3)
AE
1.2
1.0
0.8
0.6
0.4
0.2
0.0
Rxn Yield
1/SF
MRP
RME
Actual
Ideal

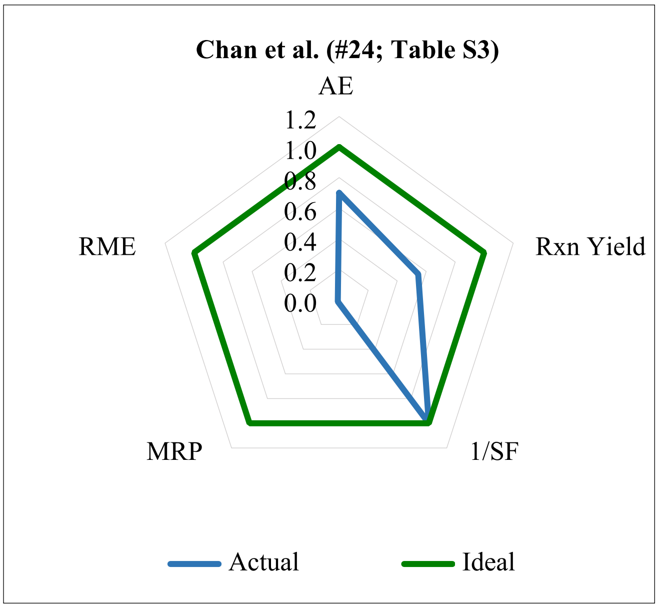

Chan et al. (#24; Table S3)
AE
1.2
1.0
0.8
0.6
0.4
0.2
0.0
Rxn Yield
1/SF
MRP
RME
Actual
Ideal

[1]NMR and ^{13}C-NMR spectra

5-hydroxymethylfurfural (HMF) in $CDCl_3$

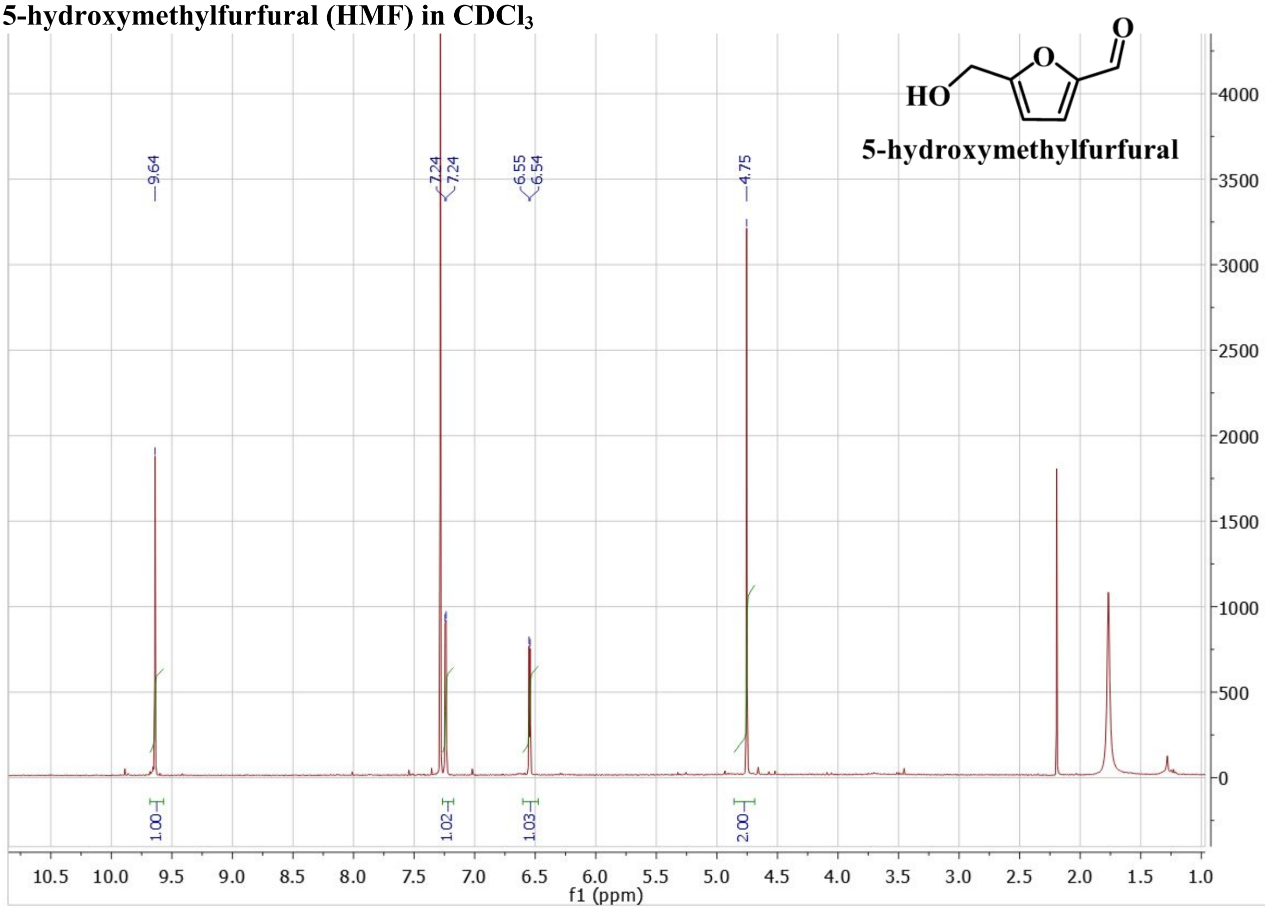

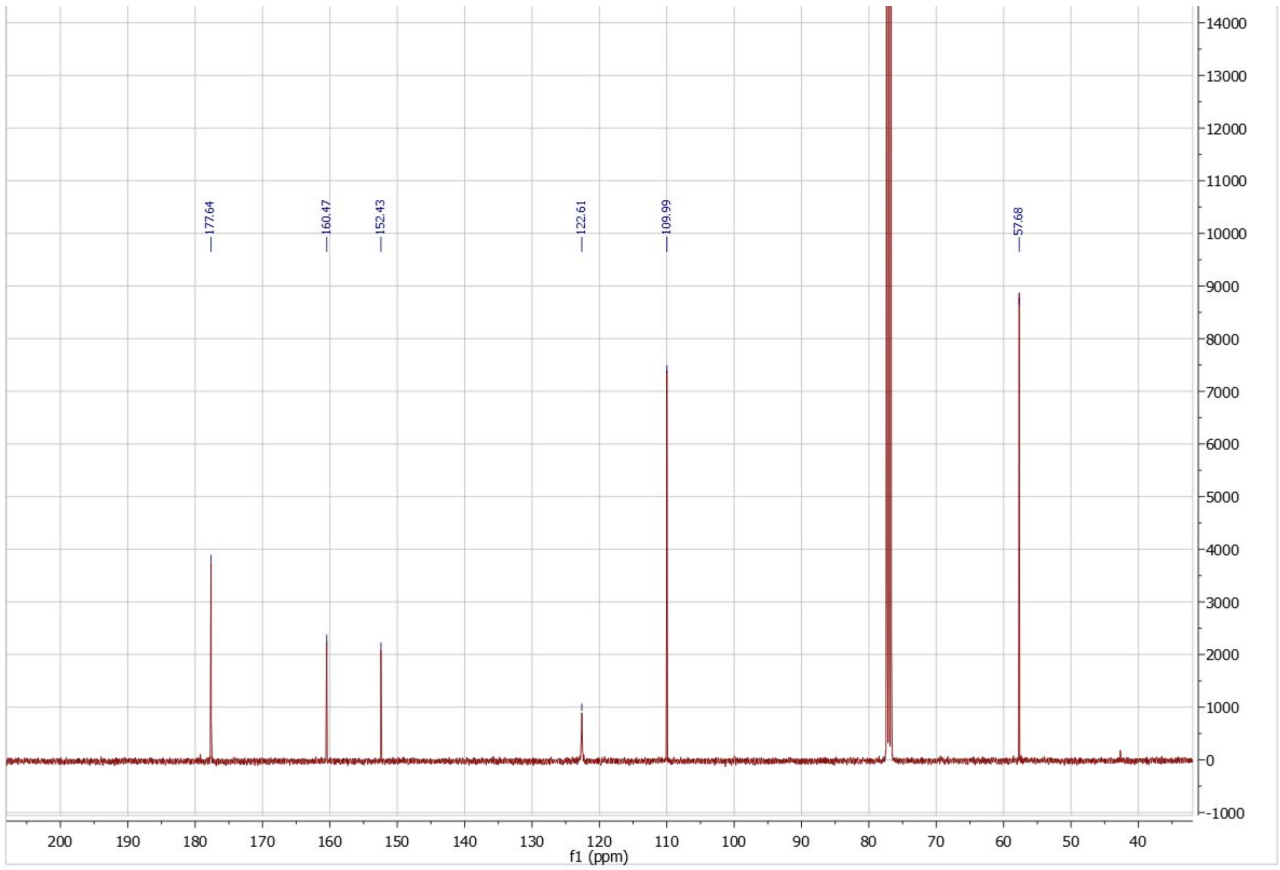

Bis-(hydroxymethyl)furan (BHMF) in MeOD

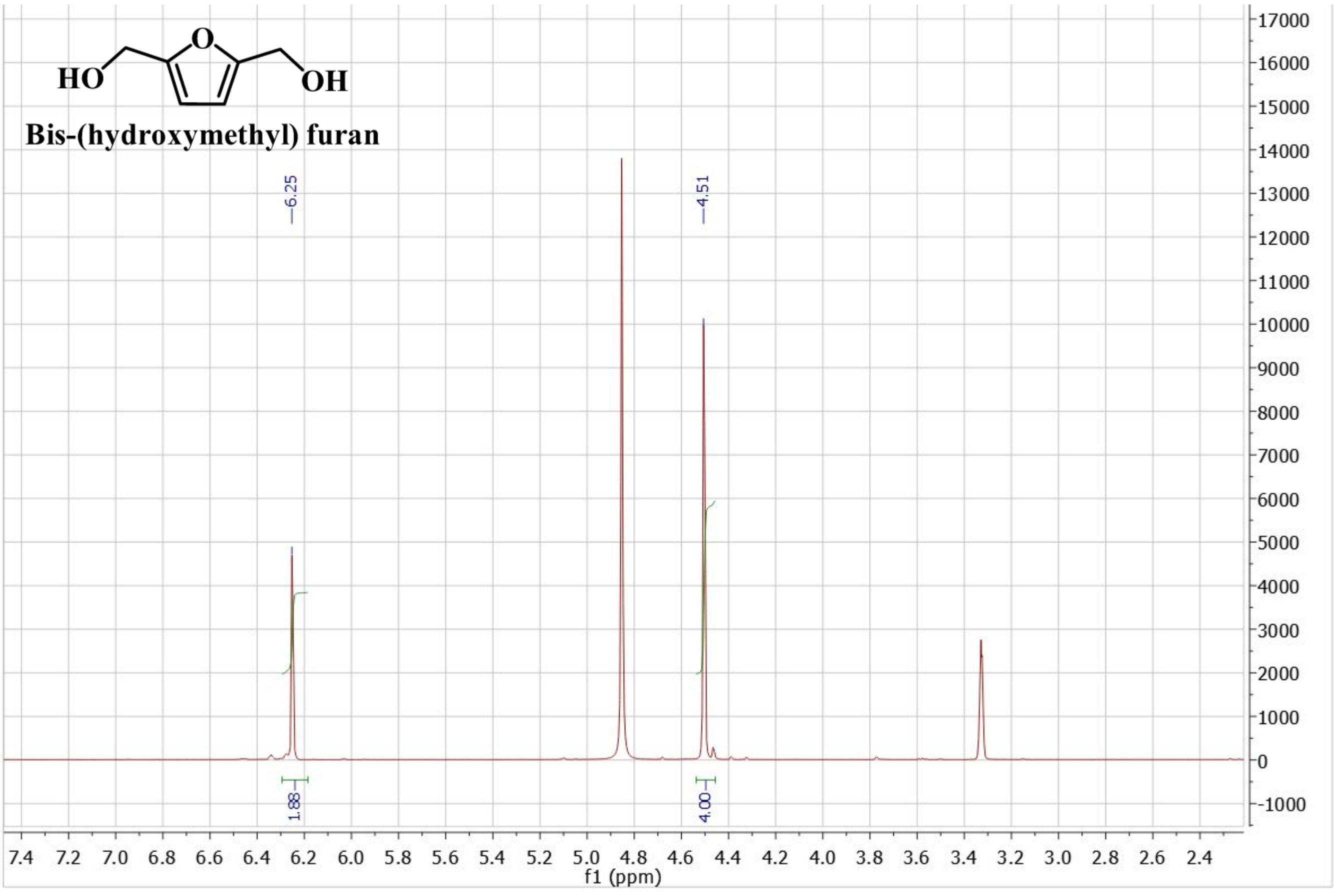

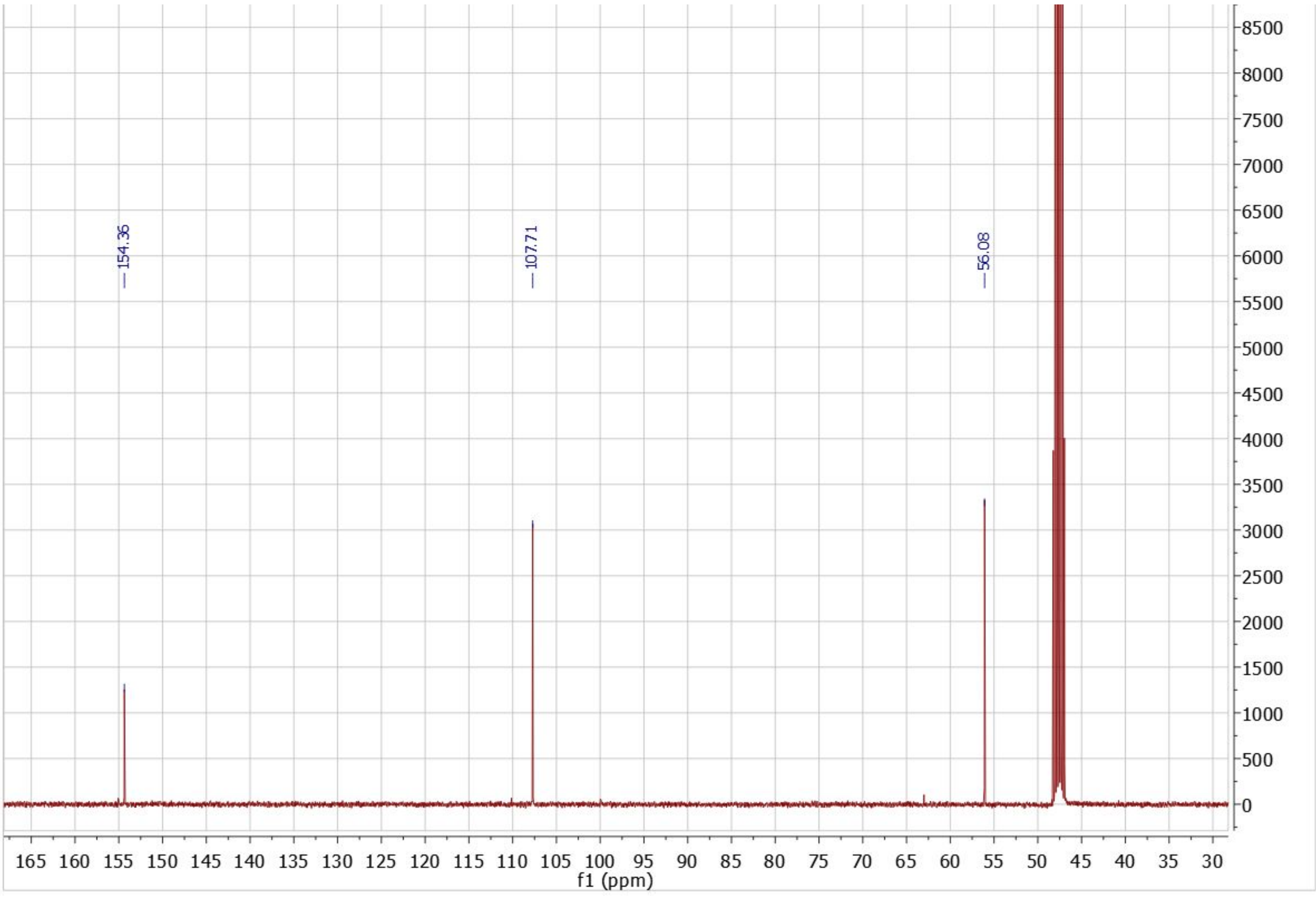

Dark-brown HMF-rich oil in $CDCl_3$

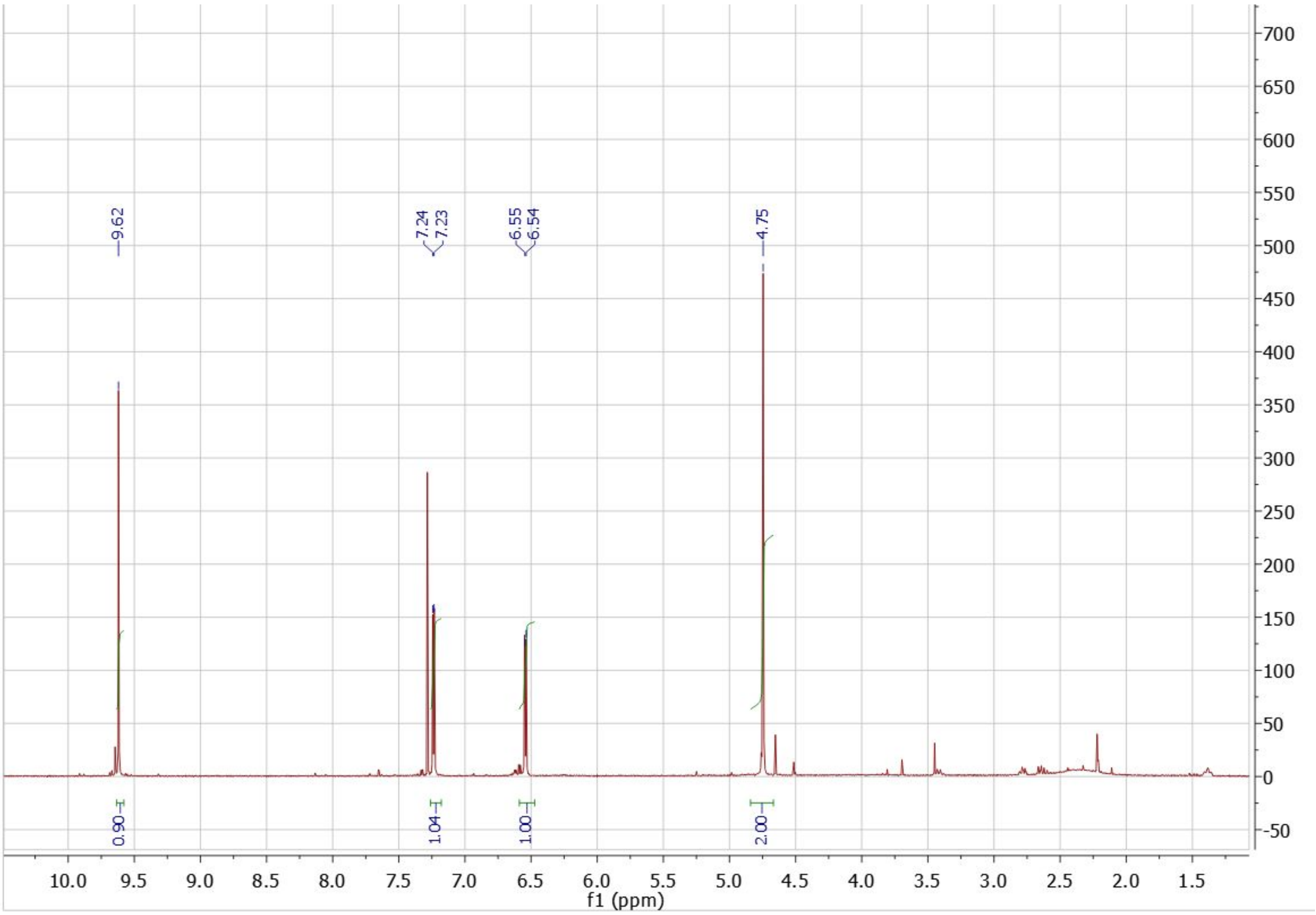

Supplement: Supplementary file 1 — op2c00196_si_001.pdf [file op2c00196_si_001.pdf]
